# From Data to Decision: Integrating Bioinformatics into Glioma Patient Stratification and Immunotherapy Selection

**DOI:** 10.3390/ijms27020667

**Published:** 2026-01-09

**Authors:** Ekaterina Sleptsova, Olga Vershinina, Mikhail Ivanchenko, Victoria Turubanova

**Affiliations:** 1 Department of Genetics and Life Sciences, Sirius University, Sochi 354340, Russia; sleptsova.ee@talantiuspeh.ru; 2 Research Center in Artificial Intelligence, Institute of Information Technologies, Mathematics and Mechanics, Lobachevsky State University, Nizhny Novgorod 603022, Russia; 3 Institute of Biogerontology, Lobachevsky State University, Nizhny Novgorod 603022, Russia; 4 Institute of Neurosciences, Lobachevsky State University, Nizhny Novgorod 603022, Russia

**Keywords:** glioblastoma (GBM), astrocytoma, oligodendroglioma, tumor microenvironment, machine learning, explainable artificial intelligence

## Abstract

Gliomas are notoriously difficult to treat owing to their pronounced heterogeneity and highly variable treatment responses. This reality drives the development of precise diagnostic and prognostic methods. This review explores the modern arsenal of bioinformatic tools aimed at refining diagnosis and stratifying glioma patients by different malignancy grades and types. We perform a comparative analysis of software solutions for processing whole-exome sequencing data, analyzing DNA methylation profiles, and interpreting transcriptomic data, highlighting their key advantages and limited applicability in routine clinical practice. Special emphasis is placed on the contribution of bioinformatics to fundamental oncology, as these tools aid in the discovery of new biomarker genes and potential targets for targeted therapy. The ninth section discusses the role of computational models in predicting immunotherapy efficacy. It demonstrates how integrative data analysis—including tumor mutational burden assessment, characterization of the tumor immune microenvironment, and neoantigen identification—can help identify patients who are most likely to respond to immune checkpoint inhibitors and other immunotherapeutic approaches. The obtained data provide compelling justification for including immunotherapy in standard glioma treatment protocols, provided that candidates are accurately selected based on comprehensive bioinformatic analysis. The tools discussed pave the way for transitioning from an empirical to a personalized approach in glioma patient management. However, we also note that this field remains largely in the preclinical research stage and has not yet revolutionized clinical practice. This review is intended for biological scientists and clinicians who find traditional bioinformatic tools difficult to use.

## 1. Introduction

Gliomas are a heterogeneous group of malignant tumors in the central nervous system. They are infiltrative by nature, invading healthy brain tissue and making complete surgical resection impossible without the risk of damaging critical brain functions. Gliomas, most notably the highly aggressive glioblastoma (GBM), exhibit high genetic heterogeneity, harbor cancer stem cells, and possess a remarkable ability to adapt [1,2,3]. These characteristics confer resistance to standard therapies such as radiation and chemotherapy [4,5,6]. Although significant progress has been made in understanding the genetics, cellular composition, and origins of gliomas [7], this knowledge has not translated into successful treatment methods [8]. The blood–brain barrier severely restricts the delivery of chemotherapeutic agents to the central nervous system. This limited access is a key reason for the poor prognosis of gliomas, which are associated with one of the lowest five-year survival rates in oncology. Notably, patients with the most aggressive form of glioblastoma, have a median overall survival that rarely exceeds 12–15 months, even with intensive combination therapy [9,10,11]. The high mortality rate of this disease underscores the urgent need for new, effective treatments.

Historically, glioma classification, beginning with the work of Kernhahn and Seidel in the 1920s and 1930s, and the World Health Organization (WHO) classifications of 2007 and 2009 (where histological criteria of cellularity, atypia, and mitosis predominated), was based primarily on morphology [12]. Since the WHO classifications of 2016 and especially 2021, the emphasis has shifted to molecular markers. According to the WHO CNS classification 2021, high-grade gliomas are divided into glioblastomas, oligodendrogliomas, and astrocytomas [13]. Oligodendrogliomas are characterized by IDH1/2 mutations in combination with complete 1p/19q codeletion, often with TERT promoter mutations and CIC/FUBP1 alterations, which results in a favorable prognosis and chemosensitivity [14]. IDH-mutated astrocytomas typically contain IDH1/2 mutations without 1p/19q codeletion, as well as frequent TP53 mutations and ATRX loss, leading to progression to secondary glioblastomas with distinct epigenetic profiles [15]. Glioblastomas, predominantly IDH-wild-type, exhibit aggressive molecular features including TERT promoter mutations, EGFR amplification, PTEN loss, and chromosome 7/10 alterations, leading to rapid growth and poor outcomes [15].

**Figure 1:**
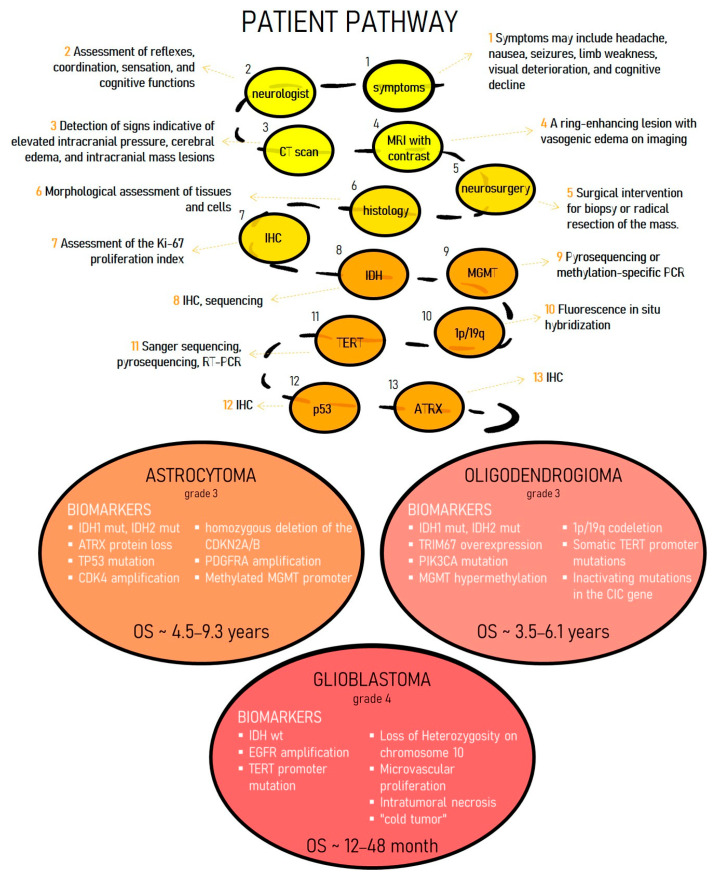
The presented diagram illustrates the extended and multi-stage diagnostic pathway of the patient with HGG. Establishing an accurate diagnosis of glioma currently represents a complex clinical challenge, which is due to the duration and significant resource intensity of the required laboratory and instrumental investigations.

Conventional diagnostic approaches for gliomas are subject to fundamental limitations that frequently result in diagnostic delays and inaccuracies. The mainstay imaging modalities include contrast-enhanced magnetic resonance imaging (MRI) and computed tomography (CT) [16]. Although MRI can visualize a tumor’s location, size, mass effect, and the contrast-enhancing ring characteristic of high-grade gliomas, it cannot reliably identify the tumor type, its molecular genetic profile, or accurately differentiate between tumor tissue and perifocal edema or nonspecific inflammation. This limitation arises because MRI detects macroscopic tissue changes but cannot reveal the tumor’s histological and biological characteristics at the cellular level. Also, traditional CT and MRI demonstrate overlapping characteristics of different glioma subtypes and limit a more detailed description of tumors at the cellular level [17,18]. Consequently, a diagnosis based solely on imaging is often preliminary and potentially inaccurate. At the same time, existing radiomics models based on MRI data of tumors demonstrate low sensitivity to glioma subtypes, especially oligodendrogliomas [19]. Consequently, a diagnosis based solely on imaging is often preliminary and potentially inaccurate. A reliable diagnosis requires histological and molecular genetic analysis of a tumor sample obtained via biopsy or resection. However, the infiltrative nature of gliomas risks sampling less aggressive tissue, which can lead to misdiagnosis and underestimation of the tumor grade [20]. This diagnostic error directly impacts treatment decisions: an incorrect assessment of the grade or subtype can result in both ineffective therapy and unnecessary overtreatment, which carries the risk of serious complications. Without precise molecular marker data, targeted therapies and immunotherapies are not feasible, leaving patients with only limited and often ineffective standard options (Figure 1) [21]. Consequently, diagnostic mistakes lead to inevitably low treatment success rates and swift disease progression.

Bioinformatic models that analyze large datasets can identify new biomarkers, build predictive models, and facilitate personalized treatment [22,23,24,25,26,27]. Existing bioinformatic models and tools for glioma classification, grading [28,29,30,31], risk groups with distinct survival outcomes and immunological characteristics [32,33,34,35] and treatment response [36,37] require a critical assessment. We must assess their clinical utility, analyze implementation challenges, and propose solutions to overcome them. This will accelerate the integration of these tools into clinical practice and ultimately improve glioma diagnostics.

This review is dedicated to assessing the practical applicability of modern bioinformatics tools for glioma classification and diagnosis, which is crucial for optimizing therapy and predicting disease outcomes. It also includes an analysis of models predicting glioma response to immunotherapy. The primary objective of this work is to evaluate the readiness of various algorithms and models for integration into routine clinical practice. To this end, we evaluate the suitability of different types of biological data (genomic, transcriptomic, etc.) for building diagnostic and prognostic systems, and analyze the effectiveness of existing models. This analysis emphasizes not only their accuracy but also such clinically significant parameters as the explainability of results and the validation of predictions.

The present review addresses bioinformatics tools underpinned by machine learning (ML), deep learning (DL), and artificial intelligence (AI) methodologies, which are designed for analyzing molecular and high-throughput biological data. To facilitate a structured and reproducible analysis, explicit literature selection criteria were defined. The inclusion criteria covered original scientific articles presenting new ML/DL/AI models developed for stratifying glioma patients, refining diagnosis, or predicting response to immunotherapy. These models were created and validated using large-scale biological datasets, such as genomic, transcriptomic, epigenomic, or integrated multimodal clinical data.

Exclusion criteria were applied to publications primarily focused on the analysis of medical images (e.g., MRI, CT, whole-slide histological images); studies based solely on traditional clinical parameters or physician notes (medical history) without integration of high-throughput molecular data; and studies limited to the validation of established molecular-genetic biomarkers (e.g., IDH mutation, MGMT promoter methylation, TERT mutation, ATRX loss) using conventional statistical methods, lacking the key component of developing an ML/AI model.

This work is intended for researchers and clinicians who find traditional bioinformatics tools difficult to use. We evaluate existing algorithms from the perspectives of both clinical data collectors and diagnosticians to determine their practical applicability.

## 2. Insufficiency of Standard Diagnostic Criteria for Glioma: Clinical Case Examples

According to the WHO 2021 classification, adult high-grade gliomas are defined by their molecular characteristics, with the IDH1/2 mutation status being the key determinant. This framework distinguishes three principal groups: (1) IDH-wildtype glioblastoma (CNS WHO grade 4), characterized by the absence of an IDH mutation and the presence of alterations such as TERT promoter mutations; (2) IDH-mutant astrocytoma (CNS WHO grade 3 or 4), defined by an IDH mutation and homozygous deletion of CDKN2A/B; and (3) IDH-mutant and 1p/19q-codeleted oligodendroglioma (CNS WHO grade 3). Contemporary diagnosis integrates histologic assessment of malignancy with molecular genetic testing to verify the tumor type and determine prognosis.

Cases of high-grade gliomas with discordant histopathological and molecular genetic findings are reported in the literature, posing a significant challenge for establishing a clear diagnosis. Discrepancies between histopathological and molecular-genetic results most often resulted in a revised tumor grade and, in some instances, a complete diagnostic reclassification. The integration of comprehensive molecular techniques, including high-throughput sequencing and DNA methylation profiling, can overcome these diagnostic difficulties. For example [38], reported a case of a 28-year-old man with a high-grade glioma harboring IDH1 and TP53 mutations, as well as co-deletion of chromosomes 1p and 19q, findings consistent with an oligodendroglioma. The diagnosis of anaplastic oligodendroglioma was further supported by histology, which showed characteristic features of this tumor type in most of the tissue sample. Following relapse, testing for MGMT promoter methylation was performed and confirmed. Further molecular testing revealed features more consistent with glioblastoma: multiple large chromosomal aberrations, including loss of the entire chromosome 1 and 2q; a missense mutation in ATRX; and amplification of MYCN, MET, and CDK4.

Case [39] involved a 72-year-old patient diagnosed with a glioma consistent with high-grade astrocytoma with piloid features (HGAP). Histologically, the tumor exhibited regional heterogeneity. Some areas contained compactly arranged, elongated spindle cells, including bizarre multinucleated forms. Other areas featured round nuclei within a fibrillar matrix, accompanied by high mitotic activity (≥6 mitoses/mm^2^), early microvascular proliferation, pseudopalisading necrosis, and pseudopapillary structures. However, methylation profiling did not align with any recognized glioma subclass. The tumor was ultimately diagnosed as a high-grade glioma (HGG) exhibiting features of both HGAP and glioblastoma, but which did not conform to any existing 2021 WHO classification of central nervous system (CNS) tumor entities.

As described in a case report [40] of a 51-year-old male, histological analysis showed a tumor exhibiting high cellularity, cortical infiltration, elevated mitotic activity, and necrosis. Immunostaining for IDH1 p.R132H was positive, and an external evaluation confirmed 1p/19q codeletion, initially supporting a diagnosis of anaplastic oligodendroglioma (WHO 2016). However, the morphological features combined with loss of ATRX expression ultimately led to a final diagnosis of glioblastoma, IDH-wildtype. This case is notable because 1p/19q codeletion and ATRX loss are typically mutually exclusive in gliomas.

A 20-year-old female patient presenting with seizures was found to have an infiltrative low-grade mass in the right frontal lobe, which was assessed as a glioma [41]. Following gross total resection, histopathological examination revealed conflicting features. The tumor (negative for IDH1 (R132H) staining, lacking 1p/19q codeletion, with preserved ATRX) exhibited moderate atypia and focal microvascular proliferation, suggestive of a high-grade glioma. However, necrosis and significant mitotic activity were absent. Initially, chemoradiation with temozolomide was recommended. To refine the diagnosis, DNA methylation profiling was performed, which identified the tumor as a dysembryoplastic neuroepithelial tumor (DNET) with no significant genetic abnormalities. Based on this result, the treatment plan was changed to active surveillance with MRI monitoring. This decision allowed the young patient to avoid radiotherapy. At the time of this case report, the patient’s condition has remained stable over 13 months of follow-up.

The same paper reports a case of a 26-year-old female presenting with seizures and an MRI-identified lesion in the left temporal lobe. The patient underwent gross total resection of the tumor. The preoperative differential diagnoses considered were astrocytoma, pleomorphic xanthoastrocytoma (PXA), or another glioma subtype. Histological examination revealed a morphology consistent with either glioblastoma or anaplastic PXA: the tumor displayed high-grade features, harbored a BRAF V600E mutation, and was negative for IDH1 (R132H). Given the diagnostic uncertainty and the presence of aggressive growth features on imaging, treatment according to the glioblastoma protocol was initiated (concurrent chemoradiotherapy followed by adjuvant temozolomide therapy). While suffering from severe depression triggered by the probable glioblastoma diagnosis, the patient considered euthanasia. To clarify the diagnosis, a tumor DNA methylation profiling test was performed. Its result, along with the analysis of chromosomal copy number variations (CNVs), confirmed the diagnosis of anaplastic PXA: a methylation class characteristic for this tumor type was identified, along with a homozygous deletion of CDKN2A and the absence of other CNV signatures typical of glioblastoma. Later, upon disease recurrence, the patient underwent a second surgery, received chemotherapy cycles, and was started on BRAF inhibitor therapy. This treatment resulted in stable disease for three years after diagnosis, and the patient no longer pursued the option of physician-assisted dying.

Collectively, these cases reveal diagnostic challenges in gliomas, where histological assessment may discord with molecular features or prove inadequate for definitive classification.

## 3. Diverse Data Sources for Constructing Classifiers

The gradual accumulation of cancer-related biological and clinical data, accelerated by advances in molecular biology and genetics, has created a substantial body of knowledge applicable to cancer diagnosis, prognosis, treatment selection, and the expansion of clinical guidelines. High-throughput sequencing has radically changed tumor diagnosis and classification, while advances in proteomics, metabolomics, and cellular immunoprofiling have deepened our understanding of tumor cell metabolism [42,43,44,45,46]. The analysis of large biological datasets requires robust bioinformatics tools, which are developing as rapidly as genetic methods themselves. Processing this data using computational and mathematical approaches must account for both the biological complexity of tumors and associated technical artifacts. Machine learning and artificial intelligence offer promising solutions to these challenges [47]. Artificial intelligence (AI) is a broad field of computer science focused on building systems that perform tasks requiring human intelligence. A pivotal subset of AI is machine learning (ML), where models are trained to identify patterns in data to perform tasks like clustering, classification, and regression [48]. The most advanced branch of ML is deep learning, which utilizes deep neural networks to automatically extract complex features from vast datasets, enabling solutions to challenging problems such as image recognition and classification [49,50]. Thus, ML models derive clinically actionable insights from diverse biomedical datasets to generate accurate predictions [51,52]. However, these advances have not yet been widely adopted in clinical practice.

Bioinformatic analysis processes a wide range of inputs, categorized by their level of biological organization: nucleic acids, proteins, metabolites, cell phenotype, tissue morphology, and physiology. The diagnostic workup of gliomas integrates imaging, histological, and molecular genetic analyses. Modern bioinformatics is increasingly centered on the analysis of genetic data, as it provides a unique window into cellular activity. For example, combining information from genomics, transcriptomics, proteomics, metabolomics, DNA methylation, and clinical parameters (e.g., survival) yields a far more complete understanding of biological data.

ML models are revolutionizing glioma diagnosis by moving beyond traditional histology to comprehensively analyze complex, large-scale molecular data. These models decipher diverse omics data to identify gene signatures and pathways linked to tumor aggressiveness, invasion, and treatment response [53,54,55,56,57]. This approach paves the way for personalized therapy and the discovery of novel therapeutic targets by pinpointing the key pathways that drive glioma progression.

The clinical translation of ML models is contingent upon their interpretability and generalizability. This requirement is a subject of intense investigation in medical research, especially in oncology, where models have achieved diagnostic accuracy on par with clinical experts [58]. At the same time, some experts argue that algorithmic transparency is not essential, as doctors often base their diagnoses on clinical experience rather than on comprehensive cause-and-effect models [59,60]. On the other hand, explainable AI methods are valued for their ability to generate new scientific insights and elucidate the connections between different pathological features. This paves the way for a new era of personalized, more effective medicine, ultimately improving patient outcomes [61].

Following a comprehensive literature review, we selected a number of scientific papers focused on the genetic and molecular prognostic modeling of glioma. A summary of the studied models is provided in Table 1, while more detailed information—including performance metrics, validation strategies, and clinical relevance—is available in Supplementary Table S1.

## 4. Leveraging NGS Data for Glioma Classification

The advent of next-generation sequencing has facilitated an explosion of omics data in cancer research, leading to significant insights into the molecular pathogenesis of diverse malignancies, from common to rare. Studies show that bioinformatics analysis of DNA sequencing data from glioma tissues allows for highly accurate molecular subtyping, with diagnostic accuracy ranging from 87% to 100% across different approaches [96,97].

Several large studies have confirmed the reliability of NGS-based panels, demonstrating that they are a robust method for detecting diagnostic DNA aberrations in gliomas with high sensitivity and specificity. In study [63], a 20-gene panel specifically tailored for gliomas was developed. All tumors were initially classified according to the 2007 WHO criteria. The gene panel was designed using the Ion AmpliSeq™ Designer tool to target previously described variants. It consisted of 660 primer pairs covering coding sequences or specific mutations in the following genes: ATRX, BRAF, CDKN2A, CDKN2B, CDKN2C, CIC, EGFR, FUBP1, H3F3A, IDH1, IDH2, NF1, NF2, NRAS, PIK3CA, PIK3R1, PTEN, RB1, TERT promoter, and TP53A. To evaluate the utility of gene panel NGS data for molecular classification, the authors performed an unsupervised hierarchical cluster analysis of 121 glioma samples. This analysis revealed clusters of genetically similar tumors that largely corresponded to the established adult glioma subtypes: IDH-wildtype glioblastoma, IDH-mutant astrocytoma, and IDH-mutant 1p/19q-codeleted oligodendroglioma.

Similar approach targeting known glioma biomarkers was employed in the study by [64]. The principle behind this approach is to consolidate all diagnostic features into a single, broad panel, eliminating the need for a battery of individual tests for each marker. Unsupervised hierarchical clustering with this panel identified three major glioma clusters and detected rare but diagnostically critical mutations, including BRAF V600E and H3F3A K27M.

The study by [98] demonstrated that NGS analysis is applicable not only to tumor tissue but also to cerebrospinal fluid (CSF). Authors analyzed mutations in IDH1, IDH2, TP53, TERT, ATRX, H3F3A, and HIST1H3B across two diffuse glioma cohorts from The Cancer Genome Atlas (TCGA), totaling 648 tumor samples. They also analyzed these seven genes in 20 clinical CSF samples from glioma patients using targeted exome sequencing and droplet digital PCR (ddPCR). Analysis of these entities led to the development of a sequencing platform for simultaneous, rapid genotyping of all seven genes in CSF-derived cell-free circulating tumor DNA (ctDNA), enabling molecular subclassification of diffuse gliomas without the need for brain tissue biopsy.

Studies that encompass the entire genomic landscape of tumors are of great significance. This approach helps expand the catalog of genomic drivers in each glioma subtype. This enables the correlation of specific tumor molecular features with patient treatment outcomes. For instance, a 2025 study [99] highlighted the potential importance of the RB1 and SMARCA4 genes in glioblastoma and the SETD2 and CREBBP genes in recurrent oligodendroglioma, suggesting an association with treatment response in these tumor types. Additional mutations were also identified in studies [100,101,102] using bioinformatics analyses.

In any discussion of glioma classification models, a recent study [69] that applies quantum computing to this task deserves particular attention. Artificial intelligence methods based on quantum computing utilize fundamental properties of quantum particles—namely superposition and quantum entanglement—to achieve massive parallel data processing. This foundational advantage translates into the ability to analyze high-dimensional data with greater efficiency and robustness against interference, qualities that are exceptionally valuable in the context of noisy medical data. The authors classified low-grade (LGG) and high-grade gliomas (HGG) from TCGA using three clinical parameters (age at diagnosis, gender, and race) and the mutation status of the 20 most common genes. The results demonstrated that the quantum model surpassed most classical machine learning algorithms in predictive accuracy.

The routine use of NGS-based models for simultaneous multi-marker evaluation provides a reliable and effective approach for comprehensive brain tumor diagnostics. The flexibility of these platforms allows for easy modification as new scientific data emerge or diagnostic criteria change. However, reducing the cost of NGS would significantly expand its use in clinical practice. We anticipate that NGS is unlikely to become part of routine glioma diagnostics in the near future.

## 5. A Methylation-Based Glioma Classifier

Sequencing data indicate that cancer initiation, progression, and maintenance result from perturbations across multiple genomic and epigenomic factors. Epigenetic modifications directly regulate the cellular transcriptomic landscape. While NGS primarily targets mutations and—depending on the technology—can simultaneously assess copy number alterations and gene fusions, there is growing interest in DNA methylation-based classification of central nervous system tumors. The diagnostic sensitivity and clinical utility of this approach have been demonstrated in a recent series of studies [103,104,105].

In 2017, a study [79] used DNA methylation data to develop signatures that enabled high-accuracy discrimination of diffuse glioma classes according to the 2016 WHO classification. Authors identified a set of 14 CpG methylation signatures via prediction analysis of microarray (PAM) that robustly differentiate LGG IDH-mutant from IDH-wildtype (WT) tumors, as validated by a support vector machine (SVM) classifier. Using similar tools, 14 CpG probes were assigned to classify oligodendrogliomas and diffuse astrocytoma and 13 CpG probes to classify IDH mutant from WT in glioblastoma.

The following year, a large-scale study [54] established a comprehensive DNA methylation-based random forest classifier for brain tumors. To evaluate the classifier’s clinical utility, the authors prospectively analyzed 1104 central nervous system tumors in parallel with standard histopathology. In 139 cases, the DNA methylation-based classification disagreed with the original pathological diagnosis. These cases were re-examined, resulting in the reclassification of the original diagnosis to align with the predicted methylation class. In particular, some IDH-WT astrocytomas and anaplastic astrocytomas were reclassified as IDH-WT glioblastomas. Beyond reclassification, the authors identified putative novel tumor variants not yet recognized by the 2016 WHO classification. Their work not only demonstrated the clinical utility of a methylation-based diagnostic tool but also highlighted its practicality for routine use. The implementation of such classification algorithms holds significant promise for standardizing tumor diagnostics across medical centers and clinical trials.

ML-based classification of CNS tumors using DNA methylation data has demonstrated high accuracy in distinguishing tumor types. A related study [86] extended this approach by analyzing methylation patterns in CSF from patients with diffuse glioma. DNA methylation profiling of CSF of patients with IDH-WT glioblastoma, and IDH-mutant diffuse gliomas, as well as non-neoplastic controls, was performed using cell-free methylated DNA immunoprecipitation and high-throughput sequencing (cfMeDIP-seq). The analysis revealed that CSF cfDNA methylation profiles exhibit distinct patterns that differentiate IDH-mutant from IDH-wildtype tumors and gliomas from controls, establishing their potential as diagnostic biomarkers.

The study published in [106] presented an approach for profiling the DNA methylation status in the serum of patients with gliomas. They identified a glioma-associated cfDNA methylation signature linked to specific immune features that effectively discriminated between patients with and without glioma based on random forest classifier. It would be interesting to determine if this approach can also distinguish between glioma subtypes, rather than merely detecting the presence of a tumor.

Although DNA methylation profiling has potential for glioma classification, its clinical adoption is hampered by several challenges. These encompass the high cost and technical complexity of the assays, the demanding requirements for high-quality and sufficient biomaterial, and the reliance on complex bioinformatics pipelines. Furthermore, key barriers include the difficulty of interpreting borderline or indeterminate results within existing classifications. Other limitations are the extended turnaround time, which precludes intraoperative use, and the high demands for computational resources and interdisciplinary expertise, all of which currently hinder the method’s widespread adoption.

## 6. The Application of Transcriptome Data for Glioma Classifier Development

Gene expression profiling captures a snapshot of a tumor’s biological phenotype at a specific point in time, providing quantitative data that reflect the tumor’s unique physiology. Significant progress has been made in using ML and other bioinformatics tools to automate the diagnosis and molecular subtyping of gliomas. Given the need to simplify this process by moving beyond the analysis of hundreds of transcripts, a variety of new assays are now being developed. The accuracy of glioma subtype classification ranged from 80.1% to 96.3% under cross-validation and from 83.3% to 93.4% in external validation. Transcriptome-based models hold high clinical significance, as they enable the identification of distinct biological subgroups with different outcomes and potential therapeutic vulnerabilities beyond standard histopathology.

One of the earliest studies to classify the most malignant gliomas—glioblastomas—was published in 2006 by a group from Genentech, Inc. [77]. Phillips et al. proposed a prognostic scheme that assigns glioblastomas to one of three subtypes (proneural, proliferative, and mesenchymal) based on their similarity to the mRNA expression signature of 35 specific genes. The prognostic value of this classification was underscored by the significant survival differences among the subtypes.

Since 2010, the development of bioinformatics models has accelerated significantly. The widespread adoption of both unsupervised (e.g., clustering) and supervised (e.g., classification) ML methods has led to more sophisticated models. These models can account for the complex biology of gliomas, particularly glioblastomas, and automatically predict tumor subtypes, independent of mutational load. Verhaak et al. proposed a classification of glioblastomas into four subtypes, which are defined by aberrations and expression profiles of the EGFR, NF1, and PDGFRA/IDH1 genes [78]. The classical subtype is defined by EGFR amplification in ~97% of cases, chromosome 7 gain/chromosome 10 loss, and PTEN deletions, correlating with mesenchymal transition and poor response to targeted therapies. The mesenchymal subtype features NF1 mutations, high expression of necrosis-associated genes like TNFRSF1A and RELB, and NF-κB pathway activation, which drive inflammation and confer relatively better immunotherapy responsiveness. The proneural subtype is enriched for PDGFRA amplification, IDH1 R132H mutations, TP53 alterations, and neuronal genes such as the SOX family, typically showing improved initial prognosis but rapid progression upon recurrence. Finally, the neural subtype expresses a normal brain glial signature with minimal defining mutations, often overlapping with oligodendroglial features and exhibiting intermediate survival outcomes [78]. Four subtypes were identified via consensus average linkage hierarchical clustering, with signature genes for each subsequently defined by a nearest centroid-based classifier (ClaNC). Consequently, a gene signature comprising 840 genes (210 per class) was established.

A comparison of the Verhaak and Phillips classification schemes shows strong agreement in assigning samples to the proneural and mesenchymal subtypes, underscoring the robustness of their respective gene signatures [107]. The Verhaak classification has long served as the primary system for diagnosing patients with different glioblastoma subtypes [108,109,110,111]. Subsequent studies, however, revealed that the neural subtype represented tumor cells contaminated with neuronal tissue [112]. This finding led to the current consensus, which recognizes only three core molecular subtypes. Moreover, in a 2019 study [80], the authors demonstrated that the original 840-gene Verhaak set, derived from a smaller number of GBM samples, could not be robustly applied to the larger TCGA cohorts now available on different platforms.

At the same time, researchers worked to classify molecular subtypes across the full spectrum of glioma malignancies. In a 2009 paper [62], the authors noted that all previous attempts to classify gliomas based on gene expression data had achieved variable success and produced inconsistent results, possibly due to the inherent bias introduced by a priori gene selection before classification. To circumvent this potential bias, the authors established a robust molecular classification of 159 gliomas by applying unsupervised ML (k-means clustering and nonnegative matrix factorization) to genome-wide expression data. The resulting model identifies two major groups, oligodendroglioma-rich and glioblastoma-rich, which are hierarchically separable into six nested subtypes.

A similar approach was used in a 2012 study [74] to complement and validate the existing molecular classification. Genome-wide gene expression profiling was performed on a cohort of 225 samples from Chinese patients. Consensus clustering revealed three main glioma groups, which were characterized by distinct survival outcomes, patient age, and IDH1 mutation frequency.

Over the past 15 years, dozens of molecular classifications for glioma have been proposed, most defining 3 to 5 subtypes. A key turning point in the development of bioinformatic models for glioma classification and predictive algorithms came after 2016. This development was directly spurred by the evolution of the WHO CNS tumor classifications. The 2016 edition marked a pivotal change by introducing molecular genetic parameters into routine diagnostics, shifting away from the purely histological foundation that had defined all previous versions. The 2007 WHO Classification of CNS Tumors utilized a purely histological approach, using microscopy and various tissue section stains for tumor assessment [13]. A 2014 meeting in the Netherlands established guidelines for integrating molecular data into brain tumor diagnosis, which subsequently formed the basis for the 2016 WHO classification. The fifth edition of the WHO classification of CNS tumors (WHO CNS5) [13] is the last version of this international standard, following prior editions published in 1979, 1993, 2000, 2007, and 2016. This paradigm shift required a fundamental change in computational tools, driving the development of bioinformatic models aligned with a new, objective, and genetically-based taxonomy. This, in turn, significantly accelerated the implementation of these models into clinical practice, as their predictions now directly operationalized the updated clinical guidelines instead of contradicting them.

The identification of new, proven important tumor-associated antigens not only expands diagnostics but can also form the basis for researching new therapeutic targets. The study [30] focused on the subclassification of gliomas into diffuse astrocytoma, anaplastic astrocytoma, and glioblastoma according to the WHO 2016 classification system. Using ML methods, the authors analyzed single-cell gene expression profiles from the Gene Expression Omnibus (GEO) dataset. This resulted in a classifier based on 539 biomarker genes that have specific expression patterns across the three subtypes. The analysis of nine most important genes (IGFBP2, IGF2BP3, PRDX1, NOV, NEFL, HOXA10, GNG12, SPRY4, and BCL11A) revealed their association with tumor growth and malignancy, cell identity and development, and cellular stress and signaling pathway regulation, thereby validating the classification model.

Gene importance analysis, applied to a classifier of three glioblastoma subtypes (classical, mesenchymal, and proneural) in study [81], revealed five genes previously unstudied in glioblastoma biology. The identified genes (NKAIN1, UBE2E2, F13A1, RNF149, and PLAUR) demonstrated a significant association of expression levels with molecular subtypes of glioblastoma and the impact on patient survival.

In study [76], the authors employed various bioinformatics approaches, such as single-sample gene set variation analysis (ssGSVA), differential gene expression (DEG) analysis, and machine learning, to establish a molecular subtyping system for gliomas founded on integrin family gene expression. The study identified two molecular subtypes, C1 and C2, which exhibited distinct malignancy grades, prognoses, and molecular and immune profiles. The C2 subtype was associated with a significantly higher prevalence of high-grade gliomas and poorer patient survival. Furthermore, molecular analysis revealed that the C2 subtype was significantly enriched for integrin genes linked to a poor prognosis, most notably ITGA7.

When discussing the heterogeneous nature of gliomas and their high invasiveness, it is important to consider that gene expression profile analyses may also include normal non-tumor cells. To address this, the study [82] developed a bioinformatics model that first identifies and excludes samples with a high normal tissue content, then classifies the remaining glioblastoma samples into three subtypes. This approach enhances diagnostic accuracy and mitigates the potential for subsequent result misinterpretation.

A team of Lithuanian researchers [83] developed classification models that stratify glioblastomas into three molecular subtypes (classical, mesenchymal, and proneural) based on the mRNA expression levels of either 5 or 20 genes. It is important to note that the authors developed primer systems for qPCR to ensure maximum data reproducibility and the application of the proposed classification models in a simpler and more accessible form in real-world settings.

Several studies have developed transcriptome-based classifiers for glioma [28,70,75] and glioblastoma [80,113] subtypes, emphasizing that therapeutic strategy should be guided by prognostic subtypes, which are strongly associated with overall survival. Specifically, additional research in a mouse model showed that the efficacy of therapeutic strategies can depend on the tumor subtype [80].

Although a variety of models can be implemented in high-tech medical centers, the latest advances in ML and cancer biology necessitate new methodologies that require fewer genes to accurately identify glioma subtypes, ultimately improving routine diagnostics.

## 7. A Multi-Dimensional View: Classifying Glioma Through an Integrative Omics Lens

Gene expression and methylation are closely interrelated processes; methylation levels in promoter regions affect gene expression by regulating the binding of transcription factors. In many cases, hypermethylation of CpG sites in promoter regions suppresses gene expression, whereas hypomethylation promotes it. Therefore, patient stratification using multiple omics data types, such as transcriptome and methylome, may provide optimal criteria for the clinical diagnosis of cancer subtypes with high accuracy [84]. The joint analysis of multiple omics modalities from the same samples holds enormous potential to advance our understanding of biological systems. To uncover a comprehensive view of tumor biology, the development and application of integrative computational methods are essential. Acknowledging these benefits, researchers have developed a range of models that integrate multiple biological datasets.

In the study [84], a biologically interpretable and highly effective model for glioblastoma subtyping was developed based on multi-omics data—the transcriptome and methylome. The results showed that models trained on transcriptome or integrated data achieved higher classification performance than those using only methylation data. Moreover, the convolutional neural network (CNN) delivered consistently high performance and surpassed traditional algorithms across all three data types. The authors emphasize that 75 marker genes derived from integrated data using the least absolute shrinkage and selection operator (LASSO) significantly improve clustering of glioblastoma subtypes compared to features derived from separate transcriptome and methylome data. Gene co-expression analysis revealed that a specific subset of features was strongly and positively correlated with a particular GBM subtype. Gene set enrichment analysis further confirmed the biological relevance of all positively correlated modules, including those associated with oncogenic processes. Additionally, the authors identified several genes within these modules that were linked to patient survival.

An alternative feature selection and data integration strategy resulted in DeepAutoGlioma, a framework that effectively diagnoses glioma malignancy subtypes (LGG and GBM) from transcriptome and methylome data [85]. The authors first identified differentially expressed genes (DEGs) and differentially methylated regions (DMRs), selecting only those associated with patient survival via univariate Cox regression for further analysis. The gene expression and methylation matrices were then input into an autoencoder, which learned the data patterns and encoded them into a low-dimensional latent space. Finally, the latent variables were used to train deep learning models for subtype classification. As a result, the CNN model demonstrated high accuracy and sensitivity, and this performance was maintained on external validation datasets.

The authors of the Portuguese study [31] used a supervised sparse canonical correlation analysis (DIABLO) to discriminate between glioma subtypes according to the WHO 2021 classification. Their models integrated multiple data modalities, including mRNA expression, DNA methylation, and miRNA expression. The work introduced two distinct models: the first classifies gliomas into three subtypes (astrocytoma, oligodendroglioma, and glioblastoma) using mRNA expression and DNA methylation data, and the second distinguishes between two LGG types (astrocytoma and oligodendroglioma) using all three designated data types. Key features with the greatest impact on subtype discrimination included the genes KIAA0495, FBXO17, C9orf64, MSN, ARSD, DRG2, THRAP3, PSMB2, NADK, PPP1R8, and methylated sites cg18222083 (TMEM106A), cg05211768 (FES), cg17105609, cg05866411 (FGFRL1), cg15603424 (ARNTL), cg24899806 (KCND2), cg19093820 (GPR156), cg26077062 (SYBU), cg04951819 (CCDC77), cg03780927 (BCL9L), and several others. Several of the identified signatures have not been previously reported in glioma research, suggesting their potential as novel biomarkers.

Study [66] developed a comprehensive framework to identify glioma-specific biomarkers, with a focus on glioblastoma. The framework employs MINGLE (Multi-omics Integrated Network for Graphical Exploration), a novel methodology that integrates disparate multi-omics data into a unified network. The analysis was conducted using transcriptomic and methylomic datasets, and patients were grouped according to the 2021 WHO glioma classification guidelines. The MINGLE networks leverage the known regulatory relationship between DNA methylation and gene expression by using genes as nodes and calculating edges based on methylomic networks. Patient classification confirmed that a subset of the identified variables can serve as biomarkers for glioma heterogeneity, providing clear distinctions between specific subtypes.

The authors of paper [71] performed a comprehensive bioinformatics analysis that integrated gene expression, whole exome sequencing, and DNA methylation data to identify prognostic factors in gliomas. The analysis first identified 17 genes associated with glioma histological grade (LGG vs. GBM) by applying differential expression analysis, and ML algorithms (LASSO and random forest). The authors then examined mutation and DNA methylation patterns, finding that methylation levels at specific CpG sites were significantly associated with IDH1 mutation status. Interestingly, some hypermethylated CpGs were more common in GBM, even though IDH1 mutations are a hallmark of LGG, suggesting complex methylation regulation. By integrating multi-omics data—genetic markers, DNA methylation, and gene expression—the authors constructed gene networks linked to tumor type determination. These networks revealed 178 prognostic genes whose activity (via DNA methylation and expression levels) influences glioma prognosis; lower activity was associated with more favorable outcomes. Among these, the role of 121 genes was consistent with previous literature, while 57 represent novel candidates for glioma research. The identified marker genes are primarily associated with regulating hypoxia, oxidative stress, and oxygen transport. Notably, some are also putative regulators of glioma development during the embryonic period. This work’s most important results are a novel stratification strategy that integrates multilayered factors and new insights into the potential basis for prognosis variability in human gliomas.

## 8. Explainable Artificial Intelligence in Glioma Classification

Advances in AI, especially in ML, have demonstrated significant potential for medical applications. However, the clinical adoption of these models is hindered by several limitations, particularly the lack of explainability in their complex decision-making processes [61,114,115,116]. AI models often function as “black boxes”, generating predictions without justification. This significantly reduces trust in such systems, as clinicians require not just a prediction from a model, but also the reasoning behind it to assess its credibility. In this regard, explainable artificial intelligence (XAI) has attracted widespread attention in healthcare in recent years [117,118,119]. As demonstrated in previous sections, a large number of studies have used ML methods to classify glioma subtypes; however, only a small number of the developed models support an explainable classification.

The authors of study [65] evaluated a range of AI decision-support software libraries, illustrating their analysis with the task of classifying gliomas into three subtypes: glioblastoma multiforme, anaplastic astrocytoma, and oligodendroglioma. Classification was carried out with a random forest algorithm, utilizing clinical variables and mutation data from the 246 most frequently mutated genes. The authors conducted a thorough systematic review, which led to the identification of the 11 most relevant libraries that implement XAI approaches. These tools were used to analyze both the global and local explainability of the classification model. Global explainability is an approach that reveals the general logic of an AI system by analyzing the overall relationships between input data and model predictions. It quantifies the cumulative contribution and importance of features for the studied cohorts, providing a high-level understanding of model behavior. Local explainability, in turn, clarifies an individual prediction for a specific patient by identifying the key factors that led to the prognosis in that particular case. Among numerous XAI methods, Shapley Additive Explanations (SHAP) is particularly notable for its ability to interpret model results both globally and locally. The study found that many key variables identified by various XAI methods are already established participants in cancer signaling pathways and common glioma biomarkers. Among the most influential mutations for predicting glioma subtype are those in CIC, IDH1, ATRX, TP53, PTEN, TERT, NF1, and EGFR. The fact that some of these markers are already incorporated into the WHO classification system underscores the reliability of the developed AI model. The explainability analysis indicated that known mutations were key drivers of the predictions. Although this does not yield novel therapeutic targets, it enhances the model’s credibility by demonstrating its basis in recognized pathophysiology, thereby fostering greater trust among clinicians.

A similar approach was used in study [68], where the authors tested various ML algorithms and XAI strategies. Their goal was to build an explainable model for classifying gliomas into two classes (LGG and GBM) based on clinical data and mutations in 20 genes. The same problem was investigated in work [72] through a methodology based on the Light Gradient Boosting Machine (LightGBM) algorithm and the SHAP explainability method. For both classification systems, the IDH1 gene mutation was the most influential feature in predicting glioma grade. Analysis of the explainability plots revealed that the models interpret the presence of a mutation as an indicator of LGG, which is consistent with medical knowledge.

XAI approaches have proven effective not just for analyzing gene mutation data, but also for interpreting transcriptomic data. Study [73] introduces a heuristic feature selection algorithm that identifies gene subsets capable of classifying glioma grades III and IV from microarray expression data. Their method utilized the expectation-maximization clustering algorithm and incorporates gene filtering based on information gain. The influence of individual selected genes on the classification was assessed using SHAP. According to global explainability analysis, the two most influential genes for grade classification were IL1R2 and ZC3HAV1.

A recent study [67] presented explainable models for glioma subtype classification and overall survival prediction based on RNA-seq data. An extensive analysis of various feature selection methods and ML algorithms resulted in the SVM model that classifies gliomas as astrocytoma, oligodendroglioma, or glioblastoma using the expression of 13 genes (TERT, NOX4, MMP9, TRIM67, ZDHHC18, HDAC1, TUBB6, ADM, NOG, CHEK2, KCNJ11, KCNIP2, and VEGFA). Using the SHAP approach, the authors not only ranked the genes by their impact on classification but also analyzed the associations between expression levels and predicted subtype in detail. In particular, it was shown that the probability of predicting a specific glioma subtype was increased by the elevated expression of distinct gene sets: astrocytoma by ZDHHC18, HDAC1, and TUBB6; oligodendroglioma by TERT, TRIM67, NOG, KCNIP2, and KCNJ11; and glioblastoma by NOX4, MMP9, TERT, VEGFA, ZDHHC18, CHEK2, and ADM.

Thus, XAI sheds light on the internal mechanisms of predictive models, provides information about the biological basis of their predictions, and thereby increases the clinical value of such systems. Now the application of XAI in glioma diagnostics is nascent but holds great potential to fuel new research and eventual clinical integration.

## 9. Machine Learning Approaches for Predicting Immunotherapy Response in Glioma

Immunotherapy has emerged as a potential new treatment modality for patients with high-grade gliomas. Phase I/II trials assessed the efficacy of vaccines designed to enhance immune activity, utilizing platforms such as lymphokine-activated killer cells, cytotoxic T cells, autologous tumor cells, or dendritic cells [120,121,122,123,124,125,126]. Dynamic, specific interactions within the tumor microenvironment continually shape the balance between immune-mediated elimination and evasion. High-grade gliomas are a compelling case in point [127]. These aggressive and invariably fatal tumors are highly resistant to radiotherapy and chemotherapy, and almost always recur following surgical resection. Situated within the immune-privileged brain, high-grade gliomas utilize a multifaceted suite of defense mechanisms to shield themselves from immune assault. Despite these challenges, achieving long-term remission remains a viable possibility [128,129].

However, to enhance the efficacy of immunotherapy, it is crucial to identify patient subgroups with greater potential to respond to specific immunotherapeutic approaches. Consequently, predictive biomarkers and response assessment systems are now under development [92,130,131,132]. We note that many studies investigating glioma susceptibility to immunotherapy have chosen a single gene variant to stratify patients into different immune phenotypes: gliomas genes associated with either immunity [33,34,87], or gliomas genes associated with immune cell infiltration [88,133] or immune cell genes from the TME [89,93,127,133]. Each of these approaches presents a distinct set of trade-offs. We have identified several models with high potential for clinical application.

In a 2022 paper [89], immunotyping of lower-grade gliomas (LGG, grade II–III) was performed. To define the immune subgroups, the authors first performed single-sample gene set enrichment analysis (ssGSEA) using the expression levels of 29 immunity-associated gene signatures and subsequently applied consensus clustering based on the ssGSEA scores. The optimal number of clusters was determined to be four, designated A, B, C, and D. Based on the degree of immune infiltration, the clusters were ranked as D (highest) followed by C, B, and A. Cluster D was designated the “immune-rich” phenotype, characterized by the highest enrichment scores and lowest tumor purity, whereas cluster A represented the opposite “immune-cold” phenotype. Immune checkpoint genes with vital roles in LGG oncogenesis and progression—including PDCD1 (PD-1), CD274 (PD-L1), PDCD1LG2 (PD-L2), CTLA4, HAVCR2, and LAG3—were most highly expressed in cluster D and least expressed in cluster A. Analysis of OS (overall survival) across the clusters revealed that high immune infiltration of LGG is associated with a poorer prognosis. Furthermore, cluster D was associated with a potential for better response to anti-PD-1 therapy, in contrast to anti-CTLA-4 therapy, for which no significant associations were found in any cluster. Weighted co-expression analysis revealed that high expression of CD28, CD8A, IFNG, IL2RA, IL7R, IL15, and PRF1 was associated with an unfavorable prognosis, whereas expression of IL2 and GZMB indicated a more favorable outcome.

The study [36] describes the development of a classification model for three glioblastoma subtypes based on the immune landscape. The analysis utilized a gene expression dataset comprising 397 samples obtained from three publicly available cohorts. Through a comprehensive literature review, the authors initially selected 109 immune signatures. Subsequently, recursive feature elimination identified a final set of 61 signatures that provided optimal classification accuracy for the SVM algorithm. Additionally, 13 immunological features were identified as prognostic indicators for overall survival in glioblastoma patients and were used to construct a risk score classifier. Analysis of immune cell infiltration differences among the three GBM subgroups revealed distinct patterns in patient survival and therapy response. The mesenchymal subtype of GBM is characterized by the highest level of immune infiltration, with a predominance of macrophages, neutrophils, dendritic cells, and an exhausted CD8^+^T cells. These tumors are characterized by activation of the TNF and NF-κB pathways, which is associated with an inflammatory and highly invasive phenotype. This subtype also exhibits an elevated immune checkpoint gene (ICG) score, reflecting the average expression of PDCD1, CD274, CTLA4, HAVCR2, LAG3, and TIGIT. MES subtype is distinguished by authors as more pro-inflammatory and invasive than others. Although this glioblastoma subtype is associated with a poorer prognosis, the efficacy of immune checkpoint blockade therapy may be high.

The study presented in [32] developed a prognostic risk model (PRM) based on immune-related DEGs to predict glioblastoma patient outcomes. The study utilized RNA-sequencing data and clinical information for GBM patients from The Cancer Genome Atlas (TCGA) and Chinese Glioma Genome Atlas (CGGA). The immune-related genes (IRGs) were sourced from the InnateDB and ImmPort databases. A predictive Cox regression model was developed based on five genes (STAT3, SEMA4F, GREM2, MDK, and SREBF1) identified by differential expression analysis, weighted gene co-expression network analysis, and LASSO regression. Correlation analysis showed a close association between PRM and clinical features as well as tumor mutation burden (TMB). Immune cell infiltration in GBM tissues was profiled using the CIBERSORT algorithm. The analysis revealed a distinct immune infiltration profile between the low- and high-risk groups of the PRM, marked by significant differences in regulatory T cells, macrophages, neutrophils, and dendritic cells. A high percentage of resting CD4^+^ memory T cells, M1 macrophages, and monocytes were positively associated with overall survival. The expression of immune checkpoint genes, which play a key role in the cellular regulation of immunity (such as CD44, IL-6, and ITGAM), was elevated in the high-risk PRM group. The validation of the model and the interpretation of its findings are consistent with established research data, confirming that this tool can be used to prognosticate the potential efficacy of immunotherapeutic strategies in glioblastoma patients.

The authors of [87] addressed the challenge of selecting neoantigens for developing a cancer vaccine and identified glioblastoma subtypes that are more likely to respond to vaccination. The strong association of six overexpressed and mutated tumor antigens (ARHGAP9, ARHGAP30, CLEC7A, MAN2B1, ARPC1B, and PLB1) with patient survival and antigen-presenting cells (APCs) infiltration in glioblastoma led the authors to identify them as promising candidates for an mRNA vaccine. The developers clustered GBM based on the expression of integrated profiles of immunologically related genes into three immune subtypes (IS1–IS3) and developed a guideline for the application of an anti-glioblastoma mRNA vaccine. Patients with the IS3 GBM subtype had the lowest overall survival compared to those with the IS1 and IS2 subtypes. The IS1 and IS2 groups exhibited the lowest numbers of regulatory immune cells and immunosuppressive APCs, leading to a survival advantage. Therefore, the use of immunotherapy in these patients may elicit a stronger immune response. The high expression of immune checkpoints (PD-1, PD-L1, LAG-3, etc.) in the IS3 GBM samples indicated severe suppression of lymphocyte activation and exhaustion of existing T cells. However, the authors conclude that the IS3 subtype was more likely to benefit from vaccination in combination with immune checkpoint blockade.

Several similar studies were published between 2023 and 2024. One such study [33] presented a tool for assessing the feasibility of checkpoint inhibitor-based immunotherapy in glioma patients across different malignancy grades, utilizing RNA expression analysis. First, DEGs were identified: 7181 genes were up-regulated and 4890 genes were down-regulated in the tumor tissues. Subsequently, an analysis of IRGs among these DEGs revealed 23 immune-associated genes that were significantly correlated with the OS of glioma patients. From these genes, 8 were identified as independent prognostic factors for glioma (FGFR1, FLT3, VTN, NR2C1, SEMA4G, CFP, S100P, CHGB). Among these, FGFR1 and CFP were classified as high-risk genes, while the others were low-risk genes. Using a risk calculation formula that incorporated these eight genes, the authors calculated an Immune-Gene-Related Prognostic Score (IGRPS) for each glioma patient and successfully stratified them into high-risk and low-risk subgroups based on the median value of IGRPS. Patients with a low IGRPS demonstrated better survival outcomes. Increased infiltration of M0/M2 macrophages and neutrophils was observed in the high-IGRPS subgroup, in contrast to monocytes and activated dendritic cells, which were more prevalent in the low-IGRPS subgroup. Consequently, patients with high macrophage or CD8^+^T cell levels had a worse prognosis, while high monocyte infiltration was associated with better survival. The authors concluded that patients with a low IGRPS may be higher-priority candidates for treatment with checkpoint inhibitors. This conclusion is consistent with the research data, and the model could be applied for glioma stratification to guide subsequent decisions on prescribing immunotherapy. Furthermore, the roles of the IGRPS genes NR2C1, SEMA4G, CFP, and CHGB in gliomas and other cancers are uncharacterized. Given their identification by the model as immunologically significant in gliomas, targeted study of these genes is warranted to help translate immunotherapy from research to clinical practice.

The authors of paper [34] developed a prognostic risk score model for glioma patients based on RNA-sequencing data that incorporates 11 genes related to the cell cycle and immune response: PLOD1, CCR5, CTSZ, ITGB2, TLR2, ASPM, GINS4, KIF14, KIF2C, KPNA2, and POLD3. A total of 5476 unique genes associated with glioma were used to construct a co-expression network using the weighted correlation network analysis (WGCNA) algorithm. As a result, four significantly correlated modules were identified, which also showed reasonable Pearson correlations with clinical characteristics including glioma grade, overall survival, and survival status. The authors then performed a filtering process, feature selection, and Cox regression analysis, which yielded a small subset of 11 key genes. Based on the expression of these genes, a prognostic signature for risk assessment was developed. High-risk scores correlated with more malignant tumors and poorer survival prognosis, and vice versa. Gene ontology (GO) and Reactome pathway analysis indicated that the identified genes are enriched in immune system processes, DNA replication, cell cycle, and division, suggesting a role in enhancing immune evasion and cell proliferation in gliomas. Consistent with previous studies, four of the 11 hub genes (CCR5, TLR2, ITGB2, and CTSZ) are implicated in tumor-associated macrophages (TAM) polarization and immune suppression in gliomas. Patients with high-risk scores exhibited a more immunosuppressive microenvironment, characterized by increased infiltration of macrophages, neutrophils, resting memory CD4^+^T cells, and dendritic cells, as well as elevated expression of immune checkpoint genes and their ligands. Moreover, a low-risk score correlated with greater infiltration of monocytes, naive B cells, activated dendritic cells, naive CD4^+^T cells, memory B cells, and activated NK cells.

In the study [90], the authors developed an artificial intelligence prognostic signature (AIPS) for GBM patients. From a univariate Cox regression analysis of six cohorts, 79 overall survival-associated genes were selected to serve as predictors in multiple machine learning models. As a result, the AIPS based on the random survival forest (RSF) algorithm showed the highest predictive quality. Based on the calculated AIPS risk scores, patients were categorized into high- and low-risk groups, where the high-risk group had a significantly poorer prognosis. The authors analyzed the most significant genes in the RSF model and found that the mRNA expression levels of CHI3L1, FKBP9, HOXD11, IMPDH1, MDK, MYBL2, SETD8, TMEM2, and UST were significantly increased in GBM, while the expression levels of EPM2A and FLRT1 were significantly decreased in GBM. The predictive value of AIPS for tumor response to immunotherapy was evaluated using the tumor immune dysfunction and exclusion (TIDE) algorithm. The researchers found that patients with a low AIPS risk score had lower TIDE scores and a higher treatment response rate than those with a high score. This suggests that a high AIPS score is associated with greater immunosuppression and immune escape. The authors also found significantly higher CD8^+^T cell infiltration in the low AIPS group, supporting the notion that these patients may be more sensitive to immunotherapy.

The identification of new molecular subtypes of glioblastoma to predict response to immunotherapy was proposed in [37]. Gene expression subtypes of GBM samples from the TCGA were identified using consensus clustering based on Bayesian non-negative matrix factorization (BayesNMF) and were compared with the Verhaak et al. classification [78]. The four subtypes identified (S1–S4) were consistent with the previous classification. Specifically, S2 was predominantly proneural, S3 was mesenchymal, and S4 was classical, while S1 was a mix of neural and classical subtypes. The ssGSEA results showed that S1 exhibited a low proliferative profile, S2 and S4 exhibited a high proliferative profile, and S3 exhibited a high immune profile. Analysis of the immune landscape showed that subtype S3 (mesenchymal) is the most promising for immunotherapy due to its high immune infiltration, confirming the findings of earlier work on the immune heterogeneity of glioblastoma subtypes [134]. According to the authors, subtype S4 (classical) has a comparable likelihood of treatment success due to its high EGFR amplification. Subtype S4 has been shown to have a significantly better prognosis with bevacizumab treatment compared to the other three subtypes.

In study [91], a machine learning model was developed for the prediction of distinct immune subtypes in glioma. To characterize the glioma immune microenvironment, the authors selected 122 immune-related metagene clusters representing eight immune cell types: B lineage, CD8^+^T cells, cytotoxic lymphocytes, monocytic lineage, myeloid dendritic cells, neutrophils, NK cells, and T cells. Unsupervised cluster analysis was then performed on patients from the CGGA and TCGA databases. The results showed that the immune microenvironment of glioma patients could be divided into four distinct subtypes, differing in survival outcomes, immunosuppressive characteristics, and tumor developmental stages. The described study correlates with the WHO 2021 classification, which divides high-grade gliomas into astrocytomas, oligodendrogliomas, and glioblastomas. IDH-mutant astrocytomas and oligodendrogliomas more frequently fall into immunoactive subtypes; IDH-wildtype glioblastomas are associated with immunosuppressive subtypes identified by this model. The mesenchymal phenotype, characteristic of aggressive GBM, dominates in the IMC group, which is closely linked to mitotic nuclear division and the process of the mitotic cell cycle. The immune subtypes proposed by the authors of the model reflect the molecular heterogeneity of gliomas. Using them for patient stratification to select immunotherapy could enhance treatment efficacy.

A 2025 study used bioinformatics analysis to identify key genes associated with GBM cell invasion and to construct a prognostic model [93]. Differentially expressed invasion-related genes (DE-IRGs) were identified in GBM compared to control samples. Subsequently, five of these genes (GZMB, COL22A1, MSTN, CRYGN, and OSMR) were selected via Cox regression to construct a prognostic risk model. The high- and low-risk groups, defined by the median risk score, showed a significant difference in overall survival. The results of qPCR showed that the four invasion-related genes (GZMB, CRYGN, OSMR and MSTN) were significantly increased in GBM tumor tissues. The authors applied the TIDE algorithm to estimate the sensitivity of all patients to immunotherapy, revealing a significant negative correlation between the model’s risk score and the observed immune response.

Researchers in China analyzed differentially expressed genes in gliomas and identified RBMS1 as a single potential predictor of immune infiltration levels [94]. Prior to this study, the role of this gene in malignant central nervous system neoplasms, particularly gliomas, had not been systematically investigated. Increased RBMS1 expression was positively correlated with increased infiltration of immunosuppressive cell subsets, including Tregs, Th1 cells, and NKT cells. RBMS1 expression was also positively correlated with multiple immune checkpoint molecules (e.g., PDCD1, CD274, CTLA4), costimulatory genes (CD28, TNFRSF9), and chemokines (CXCL9, CXCL10, CCL5), suggesting its potential involvement in orchestrating immunoregulatory signaling and immune cell recruitment. Multivariate Cox regression analysis confirmed RBMS1 as an independent prognostic factor for overall survival, in addition to WHO grade and distant metastasis. The authors validated their findings using IHC on clinical samples, showing that RBMS1 overexpression is associated with a more aggressive glioma phenotype in patients.

Since we have confirmed that inducing immunogenic cell death (ICD) is a preferred cell death modality for glioma [135], we are convinced that investigating gliomas based on the potential of ICD induction is critically important. The study [95] stratified glioma patients into two distinct prognostic groups by applying consensus clustering and nonnegative matrix factorization (NMF) to 34 ICD-associated genes. The authors then analyzed DEGs between the groups and applied Cox regression to identify prognostic genes, thereby developing a tool to calculate an ICD-related risk score (ICDS). The ICDS effectively predicted glioma patient prognosis in both the training and validation cohorts. Furthermore, high ICDS scores were associated with enhanced immune cell infiltration and elevated expression of immune checkpoint genes. These findings demonstrate that the ICD signature is closely associated with tumor heterogeneity, immune cell infiltration, immune function, and, consequently, prognosis in glioma.

## 10. Discussion

This review highlights the potential of bioinformatics to refine the diagnosis and prognosis of glioma patients. However, its widespread clinical adoption faces methodological and practical challenges.

One of the key challenges is the dynamically evolving nature of oncological classification. Most existing prognostic models are based on outdated histological classifications, such as the WHO 2007 glioma subtypes, which are typical for retrospective datasets from the TCGA, CGGA, and GEO repositories. As a result, their predictions may correlate poorly with the current glioma classification, which severely limits their clinical utility. Our analysis identified only a few studies that applied the WHO 2016 or 2021 glioma classification (see Supplementary Table S1). In the study [54], to validate the developed model, the authors used an independent prospective cohort in which the diagnosis was established according to the WHO 2016 classification. The study [30] also utilized a dataset in which the subtypes corresponded to the WHO 2016 version. To construct the models described in [79,81,82], the authors selected samples from retrospective datasets according to IDH mutation status. This approach can be interpreted as adhering to the WHO 2016 diagnostic criteria. Based on the developed clustering models [63,64], gliomas initially diagnosed according to the WHO 2007 classification were reclassified into groups based on IDH mutation and 1p/19q codeletion status. Regarding the WHO 2021 classification, only two studies [31,66] developed models using TCGA data that had been reclassified according to its criteria [136]. In a number of studies on glioma subtype clustering and risk group prediction, e.g., [34,70,74,75,91], the authors used retrospective datasets but additionally assessed associations of subgroups with IDH mutation and 1p/19q codeletion status. However, most classification models (e.g., [28,67,68,69,71,72,85]), although showing high accuracy, utilized the original histological diagnoses specified in the TCGA and CGGA datasets. Thus, the shift of glioma classification to a molecular genetic basis creates significant opportunities for integrating ML. Nevertheless, in our view, to reliably identify novel glioma markers and implement robust prognostic models in clinical practice, the development process should, where possible, include reclassifying historical diagnoses in the retrospective training datasets.

Our analysis of the scientific studies revealed a dichotomy between models based on established pre-selected gene signatures, e.g., [65,68,69,72] and those derived from data-driven feature selection (encompassing both unsupervised and supervised methods, e.g., [30,31,33,75,78,79]). The former, while biologically interpretable, are inherently limited by current knowledge and may overlook novel, clinically significant biomarkers. Therefore, bioinformatics and ML approaches that analyze the entire dataset, rather than relying on predefined markers, offer a more unbiased tool for discovering novel candidate genes and therapeutic targets. However, the high cost and validation challenges of complex, multi-omics models based on sequencing data call their clinical value into question. For most clinical centers, such approaches remain largely inaccessible. A more realistic path to implementation appears to be the development of simplified yet validated diagnostic panels (e.g., PCR-based), built upon discoveries made through complex bioinformatic models, as was done in [82,83,93]. Furthermore, we believe that models should complement, rather than replace, clinical expertise.

Numerous studies have demonstrated that machine learning can successfully predict immunotherapy outcomes [32,33,34,36,37,87,89,91]. The potential of this approach is supported by the observed statistically significant efficacy. The readiness of these methods for clinical translation, however, is highly contingent on the realities of clinical practice. Barriers to their broad implementation include data constraints, model interpretability issues, small cohort sizes, and limited options for validation. Progress in this field hinges on enhancing model reliability, deepening our understanding of the immune mechanisms behind predictions, and developing context-sensitive rather than universal solutions. Overall, ML is particularly promising for targeted immunotherapy, which integrates specific immunological markers with clinical parameters. However, this field is still in its infancy and requires further validation and standardization.

Addressing the existing challenges necessitates close collaboration among researchers and open data sharing. Indeed, it is the consolidated efforts of bioinformaticians, programmers, biologists, and clinicians that will enable the optimization of data and model combinations to improve the accuracy of tumor type determination and patient survival. At the same time, the models developed must be explainable tools that offer clinically interpretable insights, moving beyond the “black box” paradigm to earn the trust of practitioners. Among the relevant studies on glioma stratification, we identified five papers that we considered separately [65,67,68,72,73]. Their distinguishing feature is the application of XAI techniques to interpret model predictions. This focus on explainability helps elucidate the relationship between biomarkers and clinical predictions, thereby enhancing the potential clinical utility of AI diagnostic systems.

A key remaining challenge is the technical and clinical validation of all tools. This includes the insufficient external validation of many models. Specifically, approximately 40% of the analyzed studies lacked external validation (Table 1 and Table S1). Furthermore, even among the studies that report performing external validation, some do not provide the specific results of this critical analysis. As has been noted, models sometimes exhibit declining accuracy on external validation datasets, indicating their poor generalization ability [32,79,85,93]. Therefore, it becomes problematic to trust models solely on the basis of high metric values derived from cross-validation or a hold-out test set from the same dataset, without rigorous external verification. Laboratory validation using biological samples, however, is a critical step that links computational predictions to their biological context, thereby verifying the functional relevance of identified genetic variants, expression patterns, or potential biomarkers for specific research aims. Thus, validation must be performed on independent cohorts using standardized methods, and the results must be statistically robust and biologically interpretable. The inherent intratumoral heterogeneity of gliomas presents a particular challenge. Effectively accounting for it requires not only multi-omics data but also the continual expansion of public databases, which is essential for improving the accuracy and reliability of predictive algorithms.

A further limitation of models based on sequencing, transcriptomics, or methylation data is their reliance on surgically obtained tissue (biopsy or resection specimens), highlighting the need for less invasive diagnostic alternatives. We view liquid biopsy as a promising approach to addressing these challenges. The analysis of circulating tumor DNA (ctDNA), tumor-derived vesicles, and circulating tumor cells from plasma or cerebrospinal fluid (CSF) enables minimally invasive diagnosis, therapy response monitoring, and real-time tracking of tumor clonal dynamics. Ultimately, this approach could reduce the need for repeated invasive biopsies, accelerate diagnosis, and integrate personalized prognostic models into routine clinical practice for glioma patients.

Currently, there is insufficient evidence to claim that ML models are revolutionizing glioma diagnostics. Despite reports of high predictive accuracy and the discovery of new molecular markers through AI-based tools, significant gaps persist. These include a lack of external validation for these approaches, weak interpretability, a shortage of prospective clinical trials, and a dearth of implementation studies in real-world clinical practice. This indicates that the field is still in a pre-clinical stage of development and has not yet transformed clinical practice.

## 11. Conclusions

This article provides an analysis of existing bioinformatics tools developed for refining the diagnosis glioma patients. We conclude that, despite the significant potential of these methods, their broad implementation into clinical practice is limited by a number of barriers. A promising direction is the development of clinically applicable tools—interpretable, aligned with current classifications, and validated diagnostic panels—that should complement, not replace, clinical expertise.

## Figures and Tables

**Table 1:** Publications discussed in this review.

Category	Reference	Data	ML Model	Number of Features	External Validation
Main glioma subtypes (astrocytoma, oligodendroglioma, and GBM)	Li et al., 2009 [62]	Transcriptome	PAM	33–352	Yes
	Zacher et al., 2017 [63]	Genome	Clustering	20	No
	Cai et al., 2018 [30]	Transcriptome	SVM	539	No
	Petersen et al., 2019 [64]	Genome	Clustering	20	No
	Gashi et al., 2022 [65]	Genome	RF	252	No
	Vieira et al., 2024 [31]	Multi-omics	DIABLO	100	No
	Coletti et al., 2025 [66]	Multi-omics	MINGLE	Not reported	No
	Vershinina et al., 2025 [67]	Transcriptome	SVM	13	Yes
Main glioma subtypes (LGG versus GBM)	Palkar et al., 2024 [68]	Genome	XGBoost	23	No
	Akpinar et al., 2025 [69]	Genome	VQC	5	No
	Yang et al., 2025 [70]	Transcriptome	SVM	Not reported	Yes
	Li et al., 2025 [71]	Multi-omics	Clustering	178	Yes
	Noviandy et al., 2025 [72]	Genome	LightGBM	23	No
	Lin et al., 2025 [73]	Transcriptome	Genetic algorithm	Not reported	No
Glioma subtypes identified by the authors	Yan et al., 2012 [74]	Transcriptome	Clustering	1801	Yes
	Tran et al., 2020 [75]	Transcriptome	Ensemble of LSVC	168	Yes
	Han et al., 2025 [76]	Transcriptome	Clustering	26	Yes
GBM subtypes	Phillips et al., 2006 [77]	Transcriptome	Clustering	35	Yes
	Li et al., 2009 [62]	Transcriptome	PAM	33–352	Yes
	Verhaak et al., 2010 [78]	Transcriptome	ClaNC	840	Yes
	Paul et al., 2017 [79]	Methylome	SVM	13	Yes
	Teo et al., 2019 [80]	Transcriptome	Clustering	500	Yes
	Tang et al., 2021 [81]	Transcriptome	XGBoost	5	Yes
	Madurga et al., 2021 [82]	Transcriptome	ClaNC	20	Yes
	Steponaitis et al., 2022 [83]	Transcriptome	LR	5 or 20	Yes
	Munquad et al., 2022 [84]	Multi-omics	CNN	75	Yes
	Munquad et al., 2023 [85]	Multi-omics	CNN	100	Yes
	Wang et al., 2024 [86]	Methylome	RF	900	No
LGG subtypes	Li et al., 2009 [62]	Transcriptome	PAM	33–352	Yes
	Paul et al., 2017 [79]	Methylome	SVM	14	Yes
	Munquad et al., 2022 [28]	Transcriptome	SVM	178	Yes
	Munquad et al., 2023 [85]	Multi-omics	CNN	400	Yes
	Vieira et al., 2024 [31]	Multi-omics	DIABLO	89	No
Immune-related glioma subtypes or risk groups	Lin et al., 2022 [87]	Transcriptome	Clustering	1658	Yes
	Luo et al., 2022 [33]	Transcriptome	Cox regression	23	Yes
	Feng et al., 2022 [88]	Transcriptome	Clustering	34	Yes
	Zhu et al., 2022 [89]	Transcriptome	Clustering	29	Yes
	Li et al., 2022 [36]	Transcriptome	SVM	61	No
	Lin et al., 2022 [32]	Transcriptome	Cox regression	5	Yes
	Guo et al., 2024 [34]	Transcriptome	Cox regression	11	Yes
	Jiang et al., 2024 [90]	Transcriptome	RSF	79	No
	Luo et al., 2024 [37]	Transcriptome	LR	13, 14, or 17	Yes
	Yuan et al., 2024 [91]	Transcriptome	LSTM	122	No
	Tong et al., 2025 [92]	Transcriptome	Clustering	285	No
	Tian et al., 2025 [93]	Transcriptome	Cox regression	5	Yes
	Zhang et al., 2025 [94]	Transcriptome	Cox regression	1	Yes
	Li et al., 2025 [95]	Transcriptome	Cox regression	14	No

ClaNC: nearest centroid-based classifier, CNN: convolutional neural network, DIABLO: data integration analysis for biomarker discovery using latent components, LightGBM: light gradient boosting machine, LR: logistic regression, LSTM: long short-term memory network, LSVC: linear support vector classifier, MINGLE: multi-omics networks into a single graph, PAM: prediction analysis of microarray, RF: random forest, RSF: random survival forest, SVM: support vector machine, VQC: variational quantum classifier, XGBoost: extreme gradient boosting.

## Data Availability

No new data were created or analyzed in this study. Data sharing is not applicable to this article.

## Supplementary Materials

The following supporting information can be downloaded at: https://www.mdpi.com/article/10.3390/ijms27020667/s1. Refs. [137,138] are cited in the Supplemental Materials.

## Abbreviations

The following abbreviations are used in this manuscript:

AI	artificial intelligence
AIPS	artificial intelligence prognostic signature
APC	antigen-presenting cell
CGGA	Chinese Glioma Genome Atlas
CDKN	Cyclin-Dependent Kinase Inhibitor
CHGB	Chromogranin B
CLEC7A	C-type lectin domain family 7 member A
CNN	convolutional neural network
CNS	central nervous system
CSF	cerebrospinal fluid
CT	computed tomography
CTLA4	Cytotoxic T-Lymphocyte-Associated protein 4
CXCL	C-X-C motif chemokine ligand
ctDNA	circulating tumor DNA
ddPCR	droplet digital PCR
DEG	differential gene expression
DE-IRGs	differentially expressed invasion-related genes
DMRs	differentially methylated regions
DNA	deoxyribonucleic acid
EGFR	Epidermal Growth Factor Receptor
FLT3	fms-like tyrosine kinase 3
GBM	glioblastoma
GEO	Gene Expression Omnibus
GO	Gene ontology
HGAP	high-grade astrocytoma with piloid features
HGG	high-grade glioma
ICD	immunogenic cell death
ICG	immune checkpoint gene
IDH	Isocitrate Dehydrogenase
IGRPS	Immune-Gene-Related Prognostic Score
IL	Interleukin
IRGs	immune-related genes
LAG-3	Lymphocyte-Activation Gene 3
LASSO	least absolute shrinkage and selection operato
LGG	low-grade glioma
MINGLE	Multi-omics Integrated Network for Graphical Exploration
ML	machine learning
MRI	magnetic resonance imaging
mRNA	messenger ribonucleic acid
NGS	next-generation sequencing
NMF	nonnegative matrix factorization
PAM	prediction analysis of microarray
PCR	polymerase chain reaction
PD-1	Programmed Cell Death protein 1
PD-L1	Programmed Death-Ligand 1
PIK3CA	Phosphatidylinositol-4,5-bisphosphate 3-kinase catalytic subunit alpha
PRM	prognostic risk model
SHAP	Shapley Additive Explanations
ssGSVA	single-sample gene set variation analysis
SVM	support vector machine
OS	overall survival
TAM	tumor-associated macrophages
TCGA	The Cancer Genome Atlas
TIDE	tumor immune dysfunction and exclusion
TLR	Toll-like receptors
TMB	tumor mutation burden
VEGFA	vascular endothelial growth factor A
VTN	Vitronectin
XAI	explainable artificial intelligence
