# Supplementary material for: From Data to Decision: Integrating Bioinformatics into Glioma Patient Stratification and Immunotherapy Selection
Source: Int J Mol Sci. 2026 Jan 9;27(2):667. doi: 10.3390/ijms27020667 (PMC12841107; doi:10.3390/ijms27020667)
Supplement: Supplementary file 1 [file ijms-27-00667-s001.zip › ijms-4032925-supplementary.pdf]

Supplementary Table S1. Characteristics of the included studies.

**Publication, year**	**Type of data**	**Problem**	**Datasets**	**Pre-selected set of genes**	**Bioinformatics and ML methods**	**Best ML model**	**Validation**	**Best model performance**	**Clinical value**	**Correlation with the WHO classification**
[1], 2006	Transcriptome data	Clustering of HGG into three subtypes: proneural, proliferative, and mesenchymal	Main dataset: 76 cases from M.D. Anderson Cancer Center (MDA);<br><br>External validation dataset: 39 cases treated at University of California San Francisco (UCSF)	No	Feature selection: univariate Cox regression analysis;<br><br>Clustering: k-means clustering	35-gene clustering model	External validation	The prognostic value of the clustering scheme was confirmed on an external dataset	Previously undescribed prognostic subclasses of HGG are identified and discovered to resemble stages in neurogenesis	Samples were graded according to the WHO 2000 criteria
[2], 2009	Transcriptome data	Three classification problems:<br>1) O main type (oligodendroglio ma-rich) and G main type (glioblastoma-rich);<br>2) two subtypes of O main type (OA and OB);<br>3) four subtypes of G main type (GA1, GA2, GB1, and GB2)	Main dataset: 159 samples from the Hermelin Brain Tumor Center, Department of Neurology and Neurosurgery at the Henry Ford Hospital;<br><br>Test dataset: 189 samples provided from a variety of institutions;<br><br>External datasets: 341 samples from GSE4271 and TCGA	No	Clustering: k-means clustering and NMF;<br><br>Classification: PAM, a supervised ML method	From 33 to 352 unique genes in the models	10-fold cross-validation on main dataset;<br><br>external validation	10-fold cross-validation accuracy:<br>1) 96%;<br>2) 92%;<br>3) 92%.<br><br>Performance metrics for the test and external sets are not provided; however, it is claimed that the reproducibility of the hierarchically nested subtypes in the test set attests to the reliability of the classification scheme derived from the training set, and the external dataset validates the scheme and the classifiers	The application of this classification system enabled the identification of previously unrecognized prognostic groups and distinct biological pathways associated with different glioma subtypes, offering insights into pathogenesis and potential therapeutic targets	Samples were assigned the WHO 2007 glioma histopathologic subtype
[3], 2010	Transcriptome data	Classification of GBM into four subtypes: proneural, neural, classical, and mesenchymal	Main dataset: TCGA-GBM (202 samples)<br><br>External validation dataset: 260 samples from four previously published studies	No	Feature selection: selection of genes with high expression variability;<br><br>Clustering: consensus average linkage hierarchical clustering;	840-gene classifier	External validation	Prediction error: 4.6%	The importance of detecting these subtypes lies in the different therapeutic approaches that different subtypes may require	The study examined the distribution of molecular markers integrated into the WHO 2016/2021 classifications – IDH mutation status, TP53, PTEN, and EGFR

					Classification: ClaNC					mutations – across GBM subtypes
[4], 2012	Transcriptome data	Clustering of glioma into three subgroups: G1, G2, and G3	Main dataset: CGGA (225 samples)<br><br>External datasets: TCGA-GBM (202 samples) and Rembrandt (475 samples)	No	Feature selection: selection of genes with high expression variability;<br><br>Clustering: consensus average linkage clustering	1801-gene clustering model	External validation	The prognostic value of the clustering scheme was confirmed on external datasets	The results reveal that 3 main subtypes stably exist in Chinese patients with glioma. The provided clustering scheme may reflect the clinical and genetic alterations more clearly	The study examined the distribution of the key WHO 2016/2021 diagnostic markers – IDH mutation status and 1p/19q codeletion – across glioma subtypes
[5], 2017	NGS data	Clustering of glioma into three subtypes associated with gene mutations	Main dataset: The CNS tumor tissue bank at the Department of Neuropathology, Heinrich Heine University, Düsseldorf, Germany (58 fresh-frozen and 80 FFPE tumor tissue samples)	Yes, a glioma-tailored gene panel covering 660 amplicons derived from 20 genes frequently aberrant in different glioma types	Unsupervised average linkage hierarchical clustering	20-gene clustering model	Sensitivity and specificity of glioma gene panel NGS for detection of DNA sequence variants and copy number changes were validated by single gene analyses	NGS analysis identified 60 of 60 previously known genetic changes, corresponding to a sensitivity of 100%	Gene panel NGS represents a robust method for the detection of diagnostic DNA aberrations in gliomas with high sensitivity and specificity.<br>Three major clusters were identified: (i) IDH 1 or 2 mutant astrocytic gliomas with frequent ATRX and TP53 gene mutations, (ii) IDH mutant oligodendroglial tumors with 1p/19q codeletion, TERT promoter mutation and frequent CIC gene mutation, and (iii) IDH wildtype glioblastomas with frequent TERT promoter mutation, PTEN mutation and/or EGFR amplification	In this study, gliomas originally diagnosed according to the WHO 2007 classification were reclassified using the WHO 2016 criteria
[6], 2017	DNA methylation data	Three classification problems:<br>1) LGG IDH mutant versus WT;	Main dataset: combination of TCGA-LGG and TCGA-GBM (654 samples);	No	Feature selection: differential methylation analysis; PAM;<br><br>Classification: SVM classifier	1) SVM model based on 14 CpGs;<br>2) SVM model based on 14 CpGs;	Split of main dataset into training (50%) and test (50%) sets;	Accuracy:<br>1) test: 99.62%, GSE58218: 98.5%, GSE48462: 85,8%;	The methylation-based molecular profiles in combination with the WHO 2016 CNS tumor classification	The TCGA samples were segregated according to the WHO 2016 classification

		2) LGG IDH mutant 1p/19q codel versus non-codel;<br>3) GBM IDH mutant versus WT	External validation datasets: GSE58218, GSE48462 and GSE36278			3) SVM model based on 13 CpGs	external validation	2) test: 96.29%, GSE58218: 97.5%, GSE48462: 78.57%;<br>3) test: 98.36%, GSE36278: 96.10%	guidelines might be able to classify the samples more precisely	
[7], 2018	DNA methylation data	Classification of CNS tumours (82 tumour classes, including glioma subtypes, and 9 control tissue classes)	Main dataset: data of the retrospective cohort of CNS tumor samples from the National Center for Tumour Diseases in Heidelberg (GSE109381) and samples from additional centers (total = 2801 tumor samples); the prospectively assessed clinical cohort as part of the National Center for Tumour Diseases Precision Oncology Program (1155 tumor samples, GSE109381);<br><br>External validation dataset: 401 samples from 5 external centres	No	Feature selection: permutation-based variable importance measure;<br><br>Classification: RF, cassifier score calibration using L2-penalized, multinomial, LR model	10000 CpGs	3-fold cross-validation on main dataset;<br><br>external validation	Misclassification error: 4.28%, AUC: 0.99	Prospectively analysis a series of 1155 diagnostic CNS tumours in parallel with standard histopathological analyses. Revision of the initial histopathological diagnosis in 12% of all cases in favour of the predicted methylation class	Pathological diagnosis was established by current pathological standard according to the WHO 2016 classification and compared to classification by methylation profiling
[8], 2018	Transcriptome data	Classification of gliomas into three subtypes: diffuse astrocytoma, anaplastic astrocytoma, and glioblastoma	Main dataset: 6341 cells from GSE89567	No	Feature selection: MCFS and IFS;<br><br>Classification: SVM classifier	539-gene SVM model	10-fold cross-validation on main dataset	Accuracy: 96.3%, Matthews correlation coefficient: 0.889	The analysis of the nine most important genes revealed their association with tumor growth and malignancy, cell identity and development, as well as the regulation of cellular stress and signaling pathways, highlighting the clinical value of the classification model	The original WHO 2016 diagnoses from the GSE89567 datasets were used for classification

[9], 2019	NGS data	Clustering of glioma into three subtypes associated with gene mutations	Main dataset: 347 gliomas (225 analyzed prospectively, 122 analyzed retrospectively)	Yes, a targeted glioma-tailored 20-gene panel	Unsupervised hierarchical clustering	20-gene clustering model	Validation in a prospective samples	Successful prospective NGS analyses were obtained for 98% of the gliomas in daily diagnostics	Application of NGS panel sequencing improves diagnostic accuracy and is feasible in daily diagnostics. Three major clusters were identified: 1) IDH 1 or 2 mutant astrocytomas with frequent mutations in TP53 and ATRX, 2) IDH-mutant and 1p/19q-codeleted oligodendrogliomas with frequent mutations in TERT and CIC, and 3) IDH-wildtype astrocytic gliomas/glioblastomas with frequent mutations in TERT, PTEN, and TP53	In this study, gliomas originally diagnosed according to the WHO 2007 classification were reclassified using the WHO 2016 criteria
[10], 2019	Transcriptome data	Clustering of GBM into three subtypes: classical, mesenchymal and proneural/neural	Main dataset: TCGA-GBM (Affymetrix U133A, 548 samples);<br><br>Validation datasets: TCGA-GBM (Agilent Custom-Array, 588 samples; RNA-seq, 168 samples);<br><br>External validation datasets: Asian-cohorts (Affymetrix Human Gene 1.0ST-Array, 61 samples; Illumina, 52 samples; Agilent 4 × 44 K, 60 samples)	No	Feature selection: differential expression analysis;<br><br>Clustering: consensus hierarchical clustering with agglomerative average linkage	500-gene clustering model	External validation	The subtype prediction model confirmed the presence of three stable GBM subtypes in data from other platforms and other ethnic groups	The developed model enables prediction of GBM subtypes in adults across different gene profiling platforms and population groups.<br>The effect of different treatment regimens on subtype survival was investigated using the subtype-specific patient-derived orthotopic xenograft (PDOX) mice	The original histology-based WHO 2007 diagnoses from the TCGA datasets were used

[11], 2020	Transcriptome data	Clustering of glioma into four transcriptome profiles: TP1, TP2a, TP2b, and TP3	Main dataset: TCGA (1032 samples);<br><br>External validation dataset: REMBRANDT (395 samples)	No	Clustering: UMAP and DBC;<br><br>Feature selection: differential expression analysis, forward feature selection, and recursive feature elimination;<br><br>Classification: ensemble model of 1000 LSVCs	168-gene classifier	External validation	The AUC for TP1 vs rest was 0.99, TP2a vs rest was 0.76, and TP3 vs rest was 0.79. TP2b could not be assessed due to low sample count	Transcriptomic profiling provides a robust and objective method to classify gliomas with high agreement to the WHO 2016 guidelines and may provide additional survival prediction to the current methods	An analysis of the concordance between transcriptome profiles and the WHO 2016 classification was performed
[12], 2021	DNA methylation data	Classification of patients with or without glioma	Main dataset: 80 serum samples from the Department of Neurosurgery, Hermelin Brain Tumor Center (HBTC) and at the Henry Ford Health System (HFHS, Detroit, MI);<br><br>External validation dataset: 44 samples	No	Feature selection: differential methylation analysis;<br><br>Classification: RF classifier	RF model based on 476 CpGs	1000-fold random split of the main dataset into training (80%) and test (20%) sets;<br><br>external validation	Accuracy: 98%	The developed glioma-specific epigenetic liquid biopsy (GeLB) score can be used as a complementary approach to diagnose and follow up patients with glioma	The study assessed IDH mutation status, a key diagnostic criterion in the WHO 2016/2021 classifications
[13], 2021	Transcriptome data	Classification of GBM into three subtypes: classical, mesenchymal, and proneural	Main dataset: 156 samples from previously published study [14]<br><br>External dataset: McMaster cohort (57 samples)	No	Feature selection: assessment of feature importance in classifier;<br><br>Classification: XGBoost classifier	Full model: 19980-gene XGBoost model;<br><br>Reduced model: 5-gene XGBoost model (NKAIN1, UBE2E2, F13A1, RNF149, and PLAUR)	10-fold cross-validation on main dataset;<br><br>external validation	10-fold cross-validation accuracy on train set (full model): 80.12%;<br><br>10-fold cross-validation accuracy on external set (reduced model): 83.28 %	The identified genes demonstrated a significant association between their expression levels and specific molecular subtypes of GBM, as well as an impact on patient survival. These findings contribute to a better understanding of the biological mechanisms underlying GBM's inter-tumoral heterogeneity	The considered datasets contained IDH-wildtype GBMs (WHO 2016)
[15], 2021	Transcriptome data	Classification of tissues into four	Main dataset:	Yes, 150 genes from [16]	Feature selection:	20-gene classifier	Split of main dataset into	F1-score (validation set):	The model incorporates a	GBM samples meeting the

		classes: normal tissue, proneural (PN), classical (CL), and mesenchymal (ME) subtype of GBM	551 IDHWT/CIMP⁻ GBM samples from TCGA, GSE4271, GSE36245, GSE48865, GSE121720, and 555 non-tumoral brain (NTB) samples from the ALLEN Human Brain Atlas;<br><br>Histology cohort: 43 GBM IDHWT samples from HM Hospitales (Madrid, Spain), Hospital General Universitario Gregorio Marañon (Madrid, Spain), and Hospital Universitario la Fe (Valencia, Spain)		differential expression analysis;<br><br>Classification: ClaNC		training (367 tumor samples and 370 NTB samples) and validation (184 tumor samples and 185 NTB samples) sets;<br><br>validation on a histological cohort using qRT-PCR-derived expression levels for classification	0.90 (PN), 0.89 (CL), 0.89 (ME)	quality parameter that detects samples with a high content of normal tissue, preventing errors in the classification and interpretation of the results in clinical practice. In addition, the expression of 20 genes can be measured by qRT-PCR	diagnostic criteria of the WHO 2016 classification were selected from retrospective datasets
[17], 2022	Transcriptome data	Classification of GBM into three subtypes: classical, mesenchymal, and proneural	Main dataset: TCGA data from the Agilent G4502 microarray platform (419 samples);<br><br>Test datasets: TCGA data from the Affymetrix HT Human Genome U133a microarray platform (419 samples) and the Illumina HiSeq 2000 platform (122 samples);<br><br>Clinical qPCR dataset: 56 samples from the LUHS (Lithuanian University of Health Sciences) cohort	Yes, 77 genes from [1,3,14,18]	Feature selection: ANOVA, ReliefF, Gini decrease, gain ratio, FCBF and mRMR;<br><br>Classification: LR with LASSO and SVM	20- or 5-gene logistic regression models	10-fold cross-validation on main dataset;<br><br>internal validation on test sets;<br><br>validation on a cohort using qRT-PCR-derived expression levels for classification	The classification accuracy of the 20-gene and 5-gene models varied between 90.7–91% and 85.9–87.7%, respectively	The classifiers were translated to a RT-qPCR assay and validated in an independent cohort of 56 glioblastomas. The authors provide designed and tested primers and optimized qPCR conditions to maximize the reproducibility of the data	The original histology-based WHO 2007 diagnoses from the TCGA dataset were used.<br><br>A total of 56 human GBM samples from the LUHS cohort were diagnosed according to the WHO 2016 classification

[19], 2022	Transcriptome data	Classification of LGG into six subtypes: astrocytoma grade 2, astrocytoma grade 3, oligoastrocytoma grade 2, oligoastrocytoma grade 3, oligodendroglioma grade 2, and oligodendroglioma grade 3	Main dataset: UCSC Xena (281 samples);<br><br>External validation datasets: GSE74462 and GSE43378	No	Dataset balancing: oversampling technique;<br><br>Feature selection: correlation-based feature selection, SVM-RFE and Boruta;<br><br>Classification: SVM, kNN, GNB, DT, and RF	178-gene SVM model	10-fold cross-validation on main dataset;<br><br>external validation	10-fold cross-validation accuracy on main dataset: 91%;<br><br>accuracy on external validation dataset: 93.39%	The findings of the present study and ML-based framework can offer new avenues for developing subtype- and grade-specific therapeutic strategies	The original histology-based WHO 2007 diagnoses from datasets were used for classification
[20], 2022	Multi-omics data (transcriptome and methylome data)	Classification of GBM into three subtypes: mesenchymal, proneural, and classical	Main dataset: TCGA-GBM (52 samples);<br><br>External validation dataset: GSE145645 (32 samples)	No	Feature selection: LASSO;<br><br>Classification: SVM, RF, GNB, LR, kNN, and CNN	75-gene CNN model	Split of main dataset into training (70%) and test (30%) sets;<br><br>10-fold cross-validation on train set;<br><br>external validation	Accuracy (cross-validation): 98.20%;<br><br>accuracy (test set): 87.50%, AUC (test set): 0.91;<br><br>accuracy (external set): 94.48%	An integrated subsystem of transcriptome and methylome data was used to build the biologically relevant model. The authors identified the genotype–phenotype relationship of GBM subtypes and the subtype-specific predictive biomarkers for potential diagnosis and treatment	The original histology-based WHO 2007 diagnoses from the TCGA datasets were used
[21], 2022	Mutation data	Classification of glioma into three subtypes: GBM, anaplastic astrocytoma, and oligodendroglioma	Main dataset: combination of 5 studies (gbm_mayo_pdx_sarkaria_2019, gbm_tcga_pub2013, glioma_mskcc_2019, lgg_tcga, and lgg_ucsf_2014) from cbioportal (1276 samples)	Yes, a set of 6 clinical variables and mutation data for 246 genes	Classification: RF classifier;<br><br>XAI: ELI5, Dalex, SHAP, LIME, InterpretML	RF model based on 252 features	Split of main dataset into training (1020 samples) and test (256 samples) sets	Accuracy (test): 87%	Providing an explainable AI system to interpret predictions and support clinical decision making	Not reported
[22], 2022	Transcriptome data	Clustering of GBM into three immune subtypes: I1, I2, and I3	Main dataset: TCGA-GBM (167 samples);<br><br>External validation dataset: CGGA mRNAseq_325 and mRNAseq_693 (369 samples)	No	Clustering: consensus clustering	1658-gene clustering model	External validation	The results were confirmed on the external dataset	The six tumor antigens are potential targets for developing anti-GBMs mRNA vaccine, and the immunotypes can be used for evaluating	The original histology-based WHO 2007 diagnoses from the TCGA and CGGA datasets were used

									vaccination response	
[23], 2022	Transcriptome data	Stratification of glioma into two risk groups associated with prognosis and immune response	Main dataset: TCGA (670 samples);<br><br>External validation dataset: CGGA (325 samples)	No	Feature selection: differential expression analysis, WGCNA, univariate Cox regression;<br><br>Risk prediction: multivariate Cox regression	23-gene prediction model	External validation	The results were confirmed on the external dataset	The authors demonstrated that the model can independently predict the clinical prognosis as well as the immune checkpoint blockade (ICB) therapy responses of glioma patients, thus having important implications on the design of immune-based therapeutic strategies	The original histology-based WHO 2007 diagnoses from the TCGA and CGGA datasets were used
[24], 2022	Transcriptome data	Clustering of GBM to identify two immune cell infiltration clusters	Main dataset: combination of TCGA-GBM, CGGA, GSE7696, and GSE4412 (1092 samples);<br><br>External validation datasets: TCGA-BRCA, TCGA-LUAD, TCGA-BLAD, TCGA-ESCA, TCGA-LGG, and gene expression data after immunotherapy (IMvigor210, 298 urothelial cancer samples)	Yes, immune cell marker genes	Feature selection: differential expression analysis;<br><br>Clustering: consensus clustering	34-gene clustering model	External validation	The ability of these gene clusters to predict the course of other tumors has been demonstrated	This study comprehensively analyzes the immune cell infiltration pattern in GBM and sheds more light on pro-/antitumor immune modulation within GBM	The original histology-based WHO 2007 diagnoses from the glioma datasets were used
[25], 2022	Transcriptome data	Clustering of LGG into four immunotypes: A, B, C, and D	Main dataset: TCGA-LGG (481 samples);<br><br>External validation datasets: CGGA-693 (332 samples), CGGA-325 (162 samples)	Yes, 29 immunity-associated signatures	ssGSEA;<br><br>Clustering: consensus clustering by the k-means algorithm	Clustering model based on 29 signatures	External validation	The results were confirmed on validation datasets	The study may provide an immunogenomics subtyping reference for immunotherapy in LGG	The study examined the distribution of molecular markers integrated into the WHO 2016/2021 classifications – IDH mutation status, PTEN, EGFR, ATRX, and TP53

										mutations – across clusters
[26], 2022	Transcriptome data	Classification of GBM into three subtypes: classical, mesenchymal, and proneural	Main dataset: combination of TCGA-GBM, Gravendeel dataset from the GlioVis database, and the Wang dataset [27] (397 samples)	Yes, 109 immune signatures	Feature selection: SVM-RFE;<br><br>Classification: SVM, RF, XGBoost, and ANN	SVM model base on 61 signatures	10-fold cross-validation on the main dataset	Accuracy: 85.38%,<br>F1-score: 0.8525	This research could provide a theoretical basis for identifying GBM subtypes by the immune signatures, followed by the development of more effective, targeted clinical treatment strategies	The study examined the correlation of subtypes with IDH mutation status, a key diagnostic criterion in the WHO 2016/2021 classifications
[28], 2022	Transcriptome data	Stratification of GBM into two risk groups associated with prognosis and immune cell infiltration	Main dataset: TCGA-GBM (156 samples);<br><br>External validation dataset: CGGA (156 samples)	Yes, 6196 immune-related genes	Feature selection: differential expression analysis, WGCNA, LASSO;<br><br>Risk prediction: multivariate Cox regression	5-gene predictive model (STAT3, SEMA4F, GREM2, MDK, and SREBF1)	External validation	AUC (TCGA): 0.75,<br>AUC (CGGA): 0.56	The low-risk and high-risk groups exhibit significant differences with respect to immune infiltration, tumor mutation burden (TMB), and tumor immune evasion. These results provide a perspective for exploring the role of immune escape in GBM	The study examined the distribution of molecular markers integrated into the WHO 2016/2021 classifications – IDH mutation status, TP53 and EGFR mutations – across risk groups
[29], 2023	Multi-omics data (transcriptome and methylome data)	Two classification problems:<br>1) subtyping of LGG (astrocytoma, oligoastrocytoma, and oligodendroglioma);<br>2) subtyping of GBM (classical, mesenchymal, and proneural)	Main datasets: TCGA-LGG (281 samples), TCGA-GBM (52 samples);<br><br>External validation datasets: GSE74462, GSE43378, GSE129477, GSE145645, GSE128654	No	Feature selection: differential expression analysis, differential methylation analysis, univariate Cox regression analysis;<br><br>Feature transformation: autoencoder;<br><br>Classification: ANN and CNN	1) CNN model based on 400 latent variables;<br>2) CNN model based on 100 latent variables	Split of main dataset into training (70%) and test (30%) sets;<br><br>10-fold cross-validation;<br><br>external validation	Accuracy (cross-validation on train set):<br>1) 98.03%,<br>2) 94.07%;<br><br>accuracy (10-fold cross-validation on test set):<br>1) 95.23%,<br>2) 90.26%;<br><br>accuracy (10-fold cross-validation on external set):<br>1) 94.48%,<br>2) 86.41%	The development of a deep-learning framework enables the integration of multi-omics data to classify the glioma subtypes to support the clinical diagnosis. The model developed based on multi-omics data can greatly support the clinician in personalizing treatment	The original histology-based WHO 2007 diagnoses from datasets were used for classification
[30], 2024	DNA methylation data	Classification of GBM into two subtypes: IDH-mutant and IDH-wildtype	Main dataset: 54 cerebrospinal fluid samples	No	Feature selection: differential methylation analysis;<br><br>Classification: RF classifier	RF model based on 900 CpGs	Split of main dataset into training (80%) and test (20%) sets	Accuracy: 81.5%	Cerebrospinal fluid (CSF) cell-free DNA (cfDNA) methylation profiles of diffuse glioma patients exhibit	Classification of GBM was performed in accordance with the WHO 2016/2021 criteria

									differential methylation patterns between IDH-mutant and IDH-wildtype tumors, as well as between tumor and control, and could be informative biomarkers for the diagnosis of patients with diffuse gliomas	
[31], 2024	Multi-omics data (transcriptome, methylome, and miRNA expression data)	Two classification problems:<br>1) subtyping of glioma (astrocytoma, oligodendroglioma, and glioblastoma);<br>2) subtyping of LGG (astrocytoma and oligodendroglioma)	Main datasets: TCGA-LGG (414 samples), TCGA-GBM (108 samples)	No	Data integration analysis for biomarker discovery using latent components (DIABLO)	1) Classifier based on 48 genes and 52 CpGs;<br>2) Classifier based on 7 genes, 71 CpGs, and 11 miRNA variables	Split of main datasets into training (70%) and test (30%) sets	Accuracy:<br>1) 0.981;<br>2) 0.973	This comprehensive approach not only allowed a highly accurate discrimination of the different TCGA glioma patients but also presented a step forward in advancing our comprehension of the underlying molecular mechanisms driving glioma heterogeneity	For this study, TCGA glioma diagnoses were reclassified according to the WHO 2021 criteria [32] and then used in the analysis
[33], 2024	Mutation data	Classification of glioma into two subtypes: LGG and GBM	Main dataset: Glioma Grading Clinical and Mutation Features Dataset from the UCI Machine Learning Repository (889 samples)	Yes, a set of 3 clinical variables and mutation data for 20 genes	Feature selection: Pearson's correlation, mutual information, PCA;<br><br>Classification: RF, DT, LR, kNN, AdaBoost, SVM, CatBoost, LightGBM, XGBoost, ANN, and CNN;<br><br>XAI: SHAP, Eli5, LIME, QLattice	XGBoost model based on 23 features	Split of main dataset into training (80%) and test (20%) sets	Accuracy (test): 88%	Providing an explainable AI system to interpret predictions and support clinical decision making	The original histology-based WHO 2007 diagnoses from the dataset were used for classification
[34], 2024	Transcriptome data	Stratification of glioma into two risk groups associated with prognosis and immune infiltration	Main dataset: CGGA mRNAseq_693 (413 samples);<br><br>Validation dataset:	Yes, 5476 glioma-related genes	Feature selection: WGCNA, LASSO;<br><br>Risk prediction: multivariate Cox regression	11-gene prediction model	Validation on the independent dataset;<br><br>external validation	The results were confirmed on validation datasets	The study provides valuable insights into molecular mechanisms and identifying potential	The study analyzed the correlation of risk groups with IDH mutation status and 1p/19q codeletion, the

			CGGA mRNAseq_325 (325 samples);<br><br>External validation dataset: 12 clinical glioma samples from the archives of Tianjin Medical University Cancer Institute and Hospital (TMUCIH)						therapeutic targets for gliomas	key diagnostic criteria of the WHO 2016/2021 classifications
[35], 2024	Transcriptome data	Stratification of GBM into two risk groups associated with prognosis and tumor immune microenvironment (TIME)	Main dataset: combination of CGGA_301, CGGA_325, CGGA_693, GSE1304, Rembrandt_475, and TCGA-GBM (984 samples)	No	Feature selection: univariate Cox regression;<br><br>Risk prediction: RSF, step Cox, LASSO, gradient boosting machine, CoxBoost, partial least-squares regression for Cox, elastic network, ridge, survival SVM, and supervised principal component algorithm	79-gene RSF model	The predictive model was built on the combined dataset and then validated on each dataset separately	5-year AUC: 0.957, 0.973, 0.968, 0.973, 0.945, and NA for CGGA_301, CGGA_325, CGGA_693, GSE1304, Rembrandt_475, and TCGA-GBM, respectively	This study developed a reliable gene signature for predicting the prognosis of GBM patients that showed excellent predictive performance and classified GBM into different states at the immune and genomic levels	The study analyzed the correlation of risk groups with IDH mutation status and 1p/19q codeletion, the key diagnostic criteria of the WHO 2016/2021 classifications
[36], 2024	Transcriptome data, protein expression data	1) Clustering of GBM into four expression subtypes: S1, S2, S3, and S4;<br><br>2) Three classification problems:<br>2.1) S2 versus rest,<br>2.2) S3 versus rest,<br>2.3) S4 versus rest	Main dataset: TCGA-GBM (RNA-seq data for 153 samples);<br><br>External validation datasets:<br>CCLE (omics data for 47 GBM cell lines),<br>CPTAC (proteomics data for 99 GBM samples),<br>GSE84010 (GBM sequencing cohort that received immunotherapy (Temozolomide treatment))	No	Clustering: consensus clustering approach based on BayesNMF;<br><br>Feature selection: LASSO;<br><br>Classification: LR classifier	1) 400-gene clustering model;<br><br>2.1) 13-gene LR classification model,<br>2.2) 17-gene LR classification model,<br>2.3) 14-gene LR classification model	1) External validation;<br><br>2) Split of main dataset into training (80%) and test (20%) sets	1) The validity of the identified subtypes was confirmed on external datasets;<br><br>2) Accuracy (test):<br>2.1) 96.7%,<br>2.2) 86.7%,<br>2.3) 93.3%	The authors reported a new classification of GBM, which divided GBM into four subtypes, each with its own specific molecular features and showing varying degrees of response rates to immunotherapy	The study examined the distribution of molecular markers integrated into the WHO 2016/2021 classifications – IDH1 mutation status, TP53 mutation, and EGFR amplification – across GBM subtypes
[37], 2024	Transcriptome data	1) Clustering of glioma into four different	Main datasets: CGGA (693 samples),	Yes, 122 immune related metagenes	Clustering: unsupervised cluster analysis	1) Clustering model based on 122 metagenes;	1) Validation on two datasets from different centers;	1) The presence of four immune clusters is	By characterizing the glioma immune	The study examined the distribution of the

		subtypes: IM1, IM2, IM3, and IM4 in the CGGA dataset; IMA, IMB, IMC, and IMD in the TCGA dataset;<br><br>2) Four classification problems:<br>2.1) IM1/IMA versus rest,<br>2.2) IM2/IMB versus rest,<br>2.3) IM3/IMC versus rest,<br>2.4) IM4/IMD versus rest	TCGA (702 samples)		(no specific method is indicated);<br><br>Feature selection: differential expression analysis;<br><br>Classification: LR, DT, RF, MLP, and LSTM	2) LSTM model base on 122 metagenes	2) 50-fold random split of the datasets into training and test sets	confirmed in two data sets;<br><br>2) AUC:<br>2.1) CGGA (IM1): 0.94, TCGA (IMA): 0.85,<br>2.2) CGGA (IM2): 0.95, TCGA (IMB): 0.97,<br>2.3) CGGA (IM3): 1.0, TCGA (IMC): 0.91,<br>2.4) CGGA (IM4): 0.95, TCGA (IMD): 0.99	microenvironment and employing machine learning to identify immune subtypes, this study lays the groundwork for future development of tailored immunotherapy strategies	key WHO 2016/2021 diagnostic markers – IDH mutation status and 1p/19q codeletion – across glioma subtypes
[38], 2025	Mutation data	Classification of glioma into two subtypes: LGG and HGG	Main dataset: combination of TCGA-LGG and TCGA-GBM (839 samples)	Yes, a set of 3 clinical variables (age at diagnosis, gender, and race) and the mutation status of the 20 genes	Feature selection: 19 filter-based, embedded-based, and wrapper-based feature selection techniques;<br><br>Classification: VQC, kNN, SVM, DT, RF, XGBoost, and gradient boosting machine	VQC based on 5 features (IDH1, age at diagnosis, PTEN, ATRX, and EGRF)	5-fold cross-validation on main dataset	Accuracy: 83%, F1-score: 0.78	Improving glioma classification accuracy by integrating classical and quantum computing	The original histology-based WHO 2007 diagnoses from the TCGA datasets were used
[39], 2025	Transcriptome data	Clustering of glioma into two molecular subtypes: C1 and C2	Main datasets: combination of CGGA325 and CGGA693 from CGGA (1018 samples);<br><br>External datasets: CGGA301, TCGA, GEO16011, GEO18494, GEO4412	Yes, 26 integrin family genes	Clustering: NMF and Brunet algorithm	26-gene clustering model	External validation	The results showed that in the external independent datasets, the samples could also be divided into two subgroups with clear prognostic significance	The findings suggest potential molecular mechanisms underlying the association between integrin family gene expression and poor prognosis in gliomas	The original histology-based WHO 2000 and WHO 2007 diagnoses from considered datasets were used
[40], 2025	Transcriptome data	Classification of glioma into two subtypes: LGG and HGG	Main dataset: combination of the 325 and 693 datasets from the CGGA;<br><br>External validation datasets: TCGA and	No	Feature selection: differential expression analysis, NMF, WGCNA;<br><br>Classification: GLM, RF, SVM-RFE, and XGBoost	SVM classification model	Split of main dataset into training (70%) and test (30%) sets;<br><br>external validation	Not reported	The authors revealed molecular differences among gliomas and identified biomarkers with potential for clinical application.	The study examined the correlation of prognosis with IDH mutation status and 1p/19q codeletion, key diagnostic criteria in the WHO

			GSE18494							2016/2021 classifications
[41], 2025	Multi-omics data (transcriptome and methylome data)	Identification of relevant gene subnetworks associated with glioma subtypes (astrocytoma, oligodendroglioma, and GBM)	Main dataset: combination of TCGA-LGG and TCGA-GBM	No	Network design: graphical lasso (glasso), joint graphical lasso (JGL) methods	Multi-omics networks into a single graph (MINGLE)	The reliability of the JGL and glasso results was tested by performing a mathematical validation. In addition, sparse multinomial logistic regression and survival analysis models were employed to investigate the relevance of the biological information carried by the sets of network-based selected variables	Not reported	The integration of multi-omics data into a single network through MINGLE facilitated the discovery of molecular relationships that reflect glioma heterogeneity, supporting the biological interpretation	For this study, TCGA glioma diagnoses were reclassified according to the WHO 2021 criteria [32] and then used in the analysis
[42], 2025	Multi-omics data (transcriptome and methylome data)	Identification of grade-related genes (between LGG and GBM)	Main datasets: TCGA-LGG (505 samples), TCGA-GBM (146 samples)<br><br>External validation dataset: CGGA	No	Differential expression analysis, WGCNA, hierarchical clustering	178 genes	External validation	The stability of the results was confirmed on an external dataset	These results provide clues for understanding glioma prognosis and highlight some novel glioma-associated genes that warrant further investigation.	The original histology-based WHO 2007 diagnoses from the TCGA and CGGA datasets were used
[43], 2025	Mutation data	Classification of glioma into two subtypes: LGG and GBM	Main dataset: combination of TCGA-LGG and TCGA-GBM (839 samples)	Yes, a set of 3 clinical variables and mutation data for 20 genes	Classification: RF, GNB, DT, SVM, kNN, and LightGBM;<br><br>XAI: SHAP	LightGBM model based on 23 features	Split of main dataset into training (80%) and test (20%) sets	Accuracy (test): 89.88%,<br>AUC (test): 0.95	A key strength of this study lies in the application of SHAP values for model explanation, which enhances transparency and clinician trust in the model's decision-making process	The original histology-based WHO 2007 diagnoses from the TCGA datasets were used for classification
[44], 2025	Transcriptome data	Classification of glioma into two subtypes: grade III and grade IV	Main dataset: GSE4412 (85 samples)	No	Feature selection: expectation-maximization clustering algorithm, genetic feature selection algorithm;<br><br>XAI: SHAP	Discretization-based genetic feature selection algorithm	Not reported	Not reported	The current study proposes a heuristic feature selection algorithm that identifies subsets of genes to almost perfectly classify glioma grades	The original histology-based WHO 2000 diagnoses from the GSE4412 dataset were used
[45], 2025	Transcriptome data	Classification of glioma into three subtypes:	Main dataset:	No	Feature selection: mutual	13-gene SVM model (TERT, NOX4, MMP9,	5-fold cross-validation on the train set;	Balanced accuracy (cross-	The resulting explainable classification of	The original histology-based WHO 2007

		astrocytoma, oligodendroglioma, and GBM	CGGA mRNAseq_693 (398 samples)<br><br>Validation/test dataset: CGGA mRNAseq_325 (229 samples)<br><br>External validation dataset: combination of TCGA-LGG and TCGA-GBM (536 samples)		information, tuned ReliefF;<br><br>Classification: kNN, SVM, RF, ERT, XGBoost, LightGBM, CatBoost, TabNet, and GANDALF;<br><br>XAI: SHAP	TRIM67, ZDHHC18, HDAC1, TUBB6, ADM, NOG, CHEK2, KCNJ11, KCNIP2, and VEGFA)	validation on the test set;<br><br>external validation	validation): 83.7%;<br><br>balanced accuracy (test set): 81.6%, AUC (test set): 0.896;<br><br>balanced accuracy (external set): 81.6%, AUC (external set): 0.896	glioma subtypes could be useful to select a personalized treatment strategy and to improve prognosis at bedside	diagnoses from the TCGA and CGGA datasets were used for classification
[46], 2025	Transcriptome data	Clustering of GBM into two clusters with different sensitivity to immunotherapy response: cluster1 and cluster2	Main datasets: TCGA (170 samples); CGGA (693 samples), GSE13041 (267 samples)	Yes, nitrogen metabolism-related genes	Feature selection: univariate Cox regression;<br><br>Clustering: consensus clustering	285-gene clustering model	Not reported	Not reported	The collected nitrogen metabolism-related genes had good classification performance, presenting notable differences in survival rates, immune levels, gene mutations, and sensitivity to drugs between cluster1 and cluster2	The original histology-based WHO 2007 diagnoses from the datasets were used
[47], 2025	Transcriptome data	Stratification of GBM into two risk groups associated with prognosis and immune infiltration	Main dataset: TCGA-GBM (169 samples);<br><br>External validation dataset: CGGA (85 samples);<br><br>qPCR dataset (10 pairs of frozen tissue samples)	Yes, 6664 invasion-related genes	Feature selection: differential expression analysis, univariate Cox regression, LASSO;<br><br>Risk prediction: multivariate Cox regression	5-gene predictive model (GZMB, COL22A1, MSTN, CRYGN and OSMR)	External validation;<br><br>qPCR of identified genes	5-year AUC (external set): 0.689	The predicted risk subgroups were associated with different survival, immune infiltration, immune response to therapy, and drug sensitivity. The risk model constructed in this study may be involved in the treatment of GBM	The original histology-based WHO 2007 diagnoses from the TCGA and CGGA datasets were used
[48], 2025	Transcriptome data	Identification of a prognostic marker in glioma associated with immune infiltration	Main datasets: GSE43378 (50 samples) and GSE178621 (22 samples);<br><br>Clinical dataset: immunohistochemistry (IHC) data	No	Feature selection: differential expression analysis;<br><br>Risk prediction: multivariate Cox regression	RBMS1	Aberrant expression of RBMS1 was confirmed in external datasets	Not reported	RBMS1 is aberrantly overexpressed in gliomas, associated with tumor aggressiveness, immunosuppressive microenvironment	The study found a correlation between RBMS1 expression and WHO 2016 grade

			from 165 glioma samples;<br><br>External validation datasets: TCGA (156 samples) and GEPIA (163 samples)						remodeling, and poor prognosis, positioning it as a promising prognostic biomarker and therapeutic target	
[49], 2025	Transcriptome data	Stratification of glioma into two risk groups associated with immunogenic cell death (ICD)	Main datasets: CGGA_325 (train set), CGGA_693 (validation set 1), and CGGA_301 (validation set 2) from CGGA;<br><br>Clinical dataset: 95 samples from Shanxi Provincial People's Hospital (Taiyuan, China)	Yes, 34 ICD-related genes	Clustering: consensus clustering and NMF;<br><br>Feature selection: differential expression analysis, univariate Cox regression, LASSO;<br><br>Risk prediction: multivariate Cox regression	14-gene predictive model (CABP1, CACNG2, CALN1, CCL18, CD70, GABRG2, GRIN3A, KCNC2, PDZK1IP1, PRLHR, PTPRT, SERPINH1, SYCE1, TNR)	Validation on other datasets from the same repository	5-year AUC: 0.795 (validation set 1) and 0.816 (validation set 2)	The authors developed the model to evaluate the prognosis and drug response of glioma patients, and confirmed that SERPINH1 promotes the malignant phenotype of gliomas by modulating the JAK/STAT signaling pathway	The study found a correlation between the risk groups and IDH mutation status, a key diagnostic criterion in the WHO 2016/2021 classifications

AdaBoost: adaptive boosting, ANN: artificial neural network, AUC: area under the curve, BayesNMF: Bayesian non-negative matrix factorization, CatBoost: categorical boosting, GANDALF: gated adaptive network for deep automated learning of features, CGGA: Chinese Glioma Genome Atlas, ClaNC: nearest centroid-based classifier, CNN: convolutional neural network, CNS: central nervous system, DBC: density-based clustering, DNA: deoxyribonucleic acid, DT: decision tree, ERT: extremely randomized trees, FCBF: fast correlation-based filter, FFPE: formalin-fixed paraffin-embedded, GBM: glioblastoma, GLM: generalized linear model, GNB: Gaussian Naïve Bayes, HGG: high-grade glioma, IDH: isocitrate dehydrogenase, IFS: incremental feature selection, kNN: k-nearest neighbors, LASSO: least absolute shrinkage and selection operator, LGG: low-grade glioma, LightGBM: light gradient boosting machine, LIME: local interpretable model-agnostic explanations, LR: logistic regression, LSTM: long short-term memory, LSVC: linear support vector classifier, MCFS: Monte Carlo feature selection, ML: machine learning, MLP: multi-layer perceptron, mRMR: minimum redundancy maximum relevance, NGS: next generation sequencing, NMF: non-negative matrix factorization, PAM: prediction analysis of microarray, PCA: principal component analysis, qRT-PCR: quantitative reverse transcription polymerase chain reaction, RF: random forest, RNA: ribonucleic acid, RSF: random survival forest, SHAP: Shapley additive explanations, ssGSEA: single-sample gene set enrichment analysis, SVM: support vector machine, SVM-RFE: support vector machine recursive feature elimination, TabNet: tabular learning neural network, TCGA: The Cancer Genome Atlas, UMAP: uniform manifold approximation and projection, VQC: variational quantum classifier, WGCNA: weighted gene co-expression network analysis, WHO: World Health Organization, WT: wildtype, XAI: explainable artificial intelligence, XGBoost: extreme gradient boosting.

References

1. Phillips, H.S.; Kharbanda, S.; Chen, R.; Forrest, W.F.; Soriano, R.H.; Wu, T.D.; Misra, A.; Nigro, J.M.; Colman, H.; Soroceanu, L.; et al. Molecular Subclasses of High-Grade Glioma Predict Prognosis, Delineate a Pattern of Disease Progression, and Resemble Stages in Neurogenesis. *Cancer Cell* **2006**, *9*, 157–173, doi:10.1016/j.ccr.2006.02.019.
2. Li, A.; Walling, J.; Ahn, S.; Kotliarov, Y.; Su, Q.; Quezado, M.; Oberholtzer, J.C.; Park, J.; Zenklusen, J.C.; Fine, H.A. Unsupervised Analysis of Transcriptomic Profiles Reveals Six Glioma Subtypes. *Cancer Research* **2009**, *69*, 2091–2099, doi:10.1158/0008-5472.CAN-08-2100.
3. Verhaak, R.G.W.; Hoadley, K.A.; Purdom, E.; Wang, V.; Qi, Y.; Wilkerson, M.D.; Miller, C.R.; Ding, L.; Golub, T.; Mesirov, J.P.; et al. Integrated Genomic Analysis Identifies Clinically Relevant Subtypes of Glioblastoma Characterized by Abnormalities in PDGFRA, IDH1, EGFR, and NF1. *Cancer Cell* **2010**, *17*, 98–110, doi:10.1016/j.ccr.2009.12.020.
4. Yan, W.; Zhang, W.; You, G.; Zhang, J.; Han, L.; Bao, Z.; Wang, Y.; Liu, Y.; Jiang, C.; Kang, C.; et al. Molecular Classification of Gliomas Based on Whole Genome Gene Expression: A Systematic Report of 225 Samples from the Chinese Glioma Cooperative Group. *Neuro-Oncology* **2012**, *14*, 1432–1440, doi:10.1093/neuonc/nos263.
5. Zacher, A.; Kaulich, K.; Stepanow, S.; Wolter, M.; Köhrer, K.; Felsberg, J.; Malzkorn, B.; Reifenberger, G. Molecular Diagnostics of Gliomas Using Next Generation Sequencing of a Glioma-Tailored Gene Panel. *Brain Pathology* **2017**, *27*, 146–159, doi:10.1111/bpa.12367.
6. Paul, Y.; Mondal, B.; Patil, V.; Somasundaram, K. DNA Methylation Signatures for 2016 WHO Classification Subtypes of Diffuse Gliomas. *Clin Epigenet* **2017**, *9*, 32, doi:10.1186/s13148-017-0331-9.
7. Capper, D.; Jones, D.T.W.; Sill, M.; Hovestadt, V.; Schrimpf, D.; Sturm, D.; Koelsche, C.; Sahm, F.; Chavez, L.; Reuss, D.E.; et al. DNA Methylation-Based Classification of Central Nervous System Tumours. *Nature* **2018**, *555*, 469–474, doi:10.1038/nature26000.
8. Cai, Y.-D.; Zhang, S.; Zhang, Y.-H.; Pan, X.; Feng, K.; Chen, L.; Huang, T.; Kong, X. Identification of the Gene Expression Rules That Define the Subtypes in Glioma. *JCM* **2018**, *7*, 350, doi:10.3390/jcm7100350.
9. Petersen, J.K.; Boldt, H.B.; Sorensen, M.; Dahlrot, R.H.; Hansen, S.; Burton, M.; Thomassen, M.; Kruse, T.; Poulsen, F.R.; Andreasen, L.; et al. GENE-33. INTEGRATED GLIOMA DIAGNOSTICS USING TARGETED NEXT-GENERATION SEQUENCING. *Neuro-Oncology* **2019**, *21*, vi104–vi104, doi:10.1093/neuonc/noz175.435.

10. Teo, W.-Y.; Sekar, K.; Seshachalam, P.; Shen, J.; Chow, W.-Y.; Lau, C.C.; Yang, H.; Park, J.; Kang, S.-G.; Li, X.; et al. Relevance of a TCGA-Derived Glioblastoma Subtype Gene-Classifier among Patient Populations. *Sci Rep* **2019**, *9*, 7442, doi:10.1038/s41598-019-43173-y.
11. Tran, P.M.H.; Tran, L.K.H.; Nechtman, J.; Dos Santos, B.; Purohit, S.; Satter, K.B.; Dun, B.; Kolhe, R.; Sharma, S.; Bollag, R.; et al. Comparative Analysis of Transcriptomic Profile, Histology, and IDH Mutation for Classification of Gliomas. *Sci Rep* **2020**, *10*, 20651, doi:10.1038/s41598-020-77777-6.
12. Sabedot, T.S.; Malta, T.M.; Snyder, J.; Nelson, K.; Wells, M.; deCarvalho, A.C.; Mukherjee, A.; Chitale, D.A.; Mosella, M.S.; Sokolov, A.; et al. A Serum-Based DNA Methylation Assay Provides Accurate Detection of Glioma. *Neuro-Oncology* **2021**, *23*, 1494–1508, doi:10.1093/neuonc/noab023.
13. Tang, Y.; Qazi, M.A.; Brown, K.R.; Mikolajewicz, N.; Moffat, J.; Singh, S.K.; McNicholas, P.D. Identification of Five Important Genes to Predict Glioblastoma Subtypes. *Neuro-Oncology Advances* **2021**, *3*, vdab144, doi:10.1093/noajnl/vdab144.
14. Wang, Q.; Hu, B.; Hu, X.; Kim, H.; Squatrito, M.; Scarpace, L.; deCarvalho, A.C.; Lyu, S.; Li, P.; Li, Y.; et al. Tumor Evolution of Glioma-Intrinsic Gene Expression Subtypes Associates with Immunological Changes in the Microenvironment. *Cancer Cell* **2017**, *32*, 42-56.e6, doi:10.1016/j.ccell.2017.06.003.
15. Madurga, R.; García-Romero, N.; Jiménez, B.; Collazo, A.; Pérez-Rodríguez, F.; Hernández-Laín, A.; Fernández-Carballal, C.; Prat-Acín, R.; Zanin, M.; Menasalvas, E.; et al. Normal Tissue Content Impact on the GBM Molecular Classification. *Briefings in Bioinformatics* **2021**, *22*, bbaa129, doi:10.1093/bib/bbaa129.
16. Wang, Q.; Hu, B.; Hu, X.; Kim, H.; Squatrito, M.; Scarpace, L.; deCarvalho, A.C.; Lyu, S.; Li, P.; Li, Y.; et al. Tumor Evolution of Glioma-Intrinsic Gene Expression Subtypes Associates with Immunological Changes in the Microenvironment. *Cancer Cell* **2018**, *33*, 152, doi:10.1016/j.ccell.2017.12.012.
17. Steponaitis, G.; Kucinskas, V.; Golubickaite, I.; Skauminas, K.; Saudargiene, A. Glioblastoma Molecular Classification Tool Based on mRNA Analysis: From Wet-Lab to Subtype. *IJMS* **2022**, *23*, 15875, doi:10.3390/ijms232415875.
18. Sandmann, T.; Bourgon, R.; Garcia, J.; Li, C.; Cloughesy, T.; Chinot, O.L.; Wick, W.; Nishikawa, R.; Mason, W.; Henriksson, R.; et al. Patients With Proneural Glioblastoma May Derive Overall Survival Benefit From the Addition of Bevacizumab to First-Line Radiotherapy and Temozolomide: Retrospective Analysis of the AVAglio Trial. *JCO* **2015**, *33*, 2735–2744, doi:10.1200/JCO.2015.61.5005.
19. Munquad, S.; Si, T.; Mallik, S.; Li, A.; Das, A.B. Subtyping and Grading of Lower-Grade Gliomas Using Integrated Feature Selection and Support Vector Machine. *Briefings in Functional Genomics* **2022**, *21*, 408–421, doi:10.1093/bfgp/elac025.
20. Munquad, S.; Si, T.; Mallik, S.; Das, A.B.; Zhao, Z. A Deep Learning–Based Framework for Supporting Clinical Diagnosis of Glioblastoma Subtypes. *Front. Genet.* **2022**, *13*, 855420, doi:10.3389/fgene.2022.855420.
21. Gashi, M.; Vuković, M.; Jekic, N.; Thalmann, S.; Holzinger, A.; Jean-Quartier, C.; Jeanquartier, F. State-of-the-Art Explainability Methods with Focus on Visual Analytics Showcased by Glioma Classification. *BioMedInformatics* **2022**, *2*, 139–158, doi:10.3390/biomedinformatics2010009.
22. Lin, H.; Wang, K.; Xiong, Y.; Zhou, L.; Yang, Y.; Chen, S.; Xu, P.; Zhou, Y.; Mao, R.; Lv, G.; et al. Identification of Tumor Antigens and Immune Subtypes of Glioblastoma for mRNA Vaccine Development. *Front. Immunol.* **2022**, *13*, 773264, doi:10.3389/fimmu.2022.773264.
23. Luo, X.; Wang, Q.; Tang, H.; Chen, Y.; Li, X.; Chen, J.; Zhang, X.; Li, Y.; Sun, J.; Han, S. A Novel Immune Gene-Related Prognostic Score Predicts Survival and Immunotherapy Response in Glioma. *Medicina* **2022**, *59*, 23, doi:10.3390/medicina59010023.
24. Feng, P.; Li, Z.; Li, Y.; Zhang, Y.; Miao, X. Characterization of Different Subtypes of Immune Cell Infiltration in Glioblastoma to Aid Immunotherapy. *Front. Immunol.* **2022**, *13*, 799509, doi:10.3389/fimmu.2022.799509.
25. Zhu, Y.; Feng, S.; Song, Z.; Wang, Z.; Chen, G. Identification of Immunological Characteristics and Immune Subtypes Based on Single-Sample Gene Set Enrichment Analysis Algorithm in Lower-Grade Glioma. *Front. Genet.* **2022**, *13*, 894865, doi:10.3389/fgene.2022.894865.
26. Li, H.; He, J.; Li, M.; Li, K.; Pu, X.; Guo, Y. Immune Landscape-Based Machine-Learning–Assisted Subclassification, Prognosis, and Immunotherapy Prediction for Glioblastoma. *Front. Immunol.* **2022**, *13*, 1027631, doi:10.3389/fimmu.2022.1027631.
27. Wang, L.-B.; Karpova, A.; Gritsenko, M.A.; Kyle, J.E.; Cao, S.; Li, Y.; Rykunov, D.; Colaprico, A.; Rothstein, J.H.; Hong, R.; et al. Proteogenomic and Metabolomic Characterization of Human Glioblastoma. *Cancer Cell* **2021**, *39*, 509-528.e20, doi:10.1016/j.ccell.2021.01.006.
28. Lin, Z.; Wang, R.; Huang, C.; He, H.; Ouyang, C.; Li, H.; Zhong, Z.; Guo, J.; Chen, X.; Yang, C.; et al. Identification of an Immune-Related Prognostic Risk Model in Glioblastoma. *Front. Genet.* **2022**, *13*, 926122, doi:10.3389/fgene.2022.926122.
29. Munquad, S.; Das, A.B. DeepAutoGlioma: A Deep Learning Autoencoder-Based Multi-Omics Data Integration and Classification Tools for Glioma Subtyping. *BioData Mining* **2023**, *16*, 32, doi:10.1186/s13040-023-00349-7.
30. Wang, D.; Shah, M.; Arjuna, S.; Dono, A.; Patel, C.; Huse, J.; Kerrigan, B.P.; Nguyen, S.; Lang, F.; Esquenazi, Y.; et al. BIOM-67. DIFFERENTIAL DNA METHYLATION PATTERNS IN THE CSF OF PATIENTS WITH DIFFUSE GLIOMAS. *Neuro-Oncology* **2024**, *26*, viii35–viii35, doi:10.1093/neuonc/noae165.0139.
31. Vieira, F.G.; Bispo, R.; Lopes, M.B. Integration of Multi-Omics Data for the Classification of Glioma Types and Identification of Novel Biomarkers. *Bioinform Biol Insights* **2024**, *18*, 11779322241249563, doi:10.1177/11779322241249563.
32. De Mendonça, M.L.; Coletti, R.; Gonçalves, C.S.; Martins, E.P.; Costa, B.M.; Vinga, S.; Lopes, M.B. Updating TCGA Glioma Classification through Integration of Molecular Profiling Data Following the 2016 and 2021 WHO Guidelines 2023.
33. Palkar, A.; Dias, C.C.; Chadaga, K.; Sampathila, N. Empowering Glioma Prognosis With Transparent Machine Learning and Interpretative Insights Using Explainable AI. *IEEE Access* **2024**, *12*, 31697–31718, doi:10.1109/ACCESS.2024.3370238.
34. Guo, K.; Yang, J.; Jiang, R.; Ren, X.; Liu, P.; Wang, W.; Zhou, S.; Wang, X.; Ma, L.; Hu, Y. Identification of Key Immune and Cell Cycle Modules and Prognostic Genes for Glioma Patients through Transcriptome Analysis. *Pharmaceuticals* **2024**, *17*, 1295, doi:10.3390/ph17101295.
35. Jiang, Q.; Yang, X.; Deng, T.; Yan, J.; Guo, F.; Mo, L.; An, S.; Huang, Q. Comprehensive Machine Learning-Based Integration Develops a Novel Prognostic Model for Glioblastoma. *Molecular Therapy: Oncology* **2024**, *32*, 200838, doi:10.1016/j.omton.2024.200838.
36. Luo, D.; Luo, A.; Hu, S.; Ye, G.; Li, D.; Zhao, H.; Peng, B. Genomics and Proteomics to Determine Novel Molecular Subtypes and Predict the Response to Immunotherapy and the Effect of Bevacizumab in Glioblastoma. *Sci Rep* **2024**, *14*, 17630, doi:10.1038/s41598-024-68648-5.
37. Yuan, F.; Wang, Y.; Yuan, L.; Ye, L.; Hu, Y.; Cheng, H.; Li, Y. Machine Learning–Based New Classification for Immune Infiltration of Gliomas. *PLoS ONE* **2024**, *19*, e0312071, doi:10.1371/journal.pone.0312071.
38. Akpinar, E.; Oduncuoglu, M. Hybrid Classical and Quantum Computing for Enhanced Glioma Tumor Classification Using TCGA Data. *Sci Rep* **2025**, *15*, 25935, doi:10.1038/s41598-025-97067-3.
39. Han, H.; Feng, P.; Yuan, G. Molecular Typing of Gliomas on the Basis of Integrin Family Genes and a Functional Study of ITGA7. *Sci Rep* **2025**, *15*, 12306, doi:10.1038/s41598-025-97342-3.
40. Yang, P.; Feng, P.; Tian, G.; Zhao, G.; Yuan, G.; Pan, Y. Integrative Machine Learning and Bioinformatics Analysis Unveil Key Genes for Precise Glioma Classification and Prognosis Evaluation. *Computational Biology and Chemistry* **2025**, *119*, 108510, doi:10.1016/j.compbiolchem.2025.108510.

41. Coletti, R.; Carrilho, J.F.; Martins, E.P.; Gonçalves, C.S.; Costa, B.M.; Lopes, M.B. A Novel Tool for Multi-Omics Network Integration and Visualization: A Study of Glioma Heterogeneity. *Computers in Biology and Medicine* **2025**, *188*, 109811, doi:10.1016/j.compbiomed.2025.109811.
42. Li, Y.; Sun, H. Multi-Omics Analysis Identifies Novels Genes Involved in Glioma Prognosis. *Sci Rep* **2025**, *15*, 5806, doi:10.1038/s41598-025-90658-0.
43. Noviandy, T.R.; Idroes, G.M.; Hardi, I. Integrating Explainable Artificial Intelligence and Light Gradient Boosting Machine for Glioma Grading. *Informatics and Health* **2025**, *2*, 1–8, doi:10.1016/j.infoh.2024.12.001.
44. Lin, T.-H.; Lin, H.-Y. Genetic Feature Selection Algorithm as an Efficient Glioma Grade Classifier. *Sci Rep* **2025**, *15*, 15497, doi:10.1038/s41598-024-83879-2.
45. Vershinina, O.; Turubanova, V.; Krivonosov, M.; Trukhanov, A.; Ivanchenko, M. Explainable Machine Learning Models for Glioma Subtype Classification and Survival Prediction. *Cancers* **2025**, *17*, 2614, doi:10.3390/cancers17162614.
46. Tong, M.; Xu, Z.; Wang, L.; Chen, H.; Wan, X.; Xu, H.; Yang, S.; Tu, Q. An Analysis of Prognostic Risk and Immunotherapy Response of Glioblastoma Patients Based on Single-Cell Landscape and Nitrogen Metabolism. *Neurobiology of Disease* **2025**, *211*, 106935, doi:10.1016/j.nbd.2025.106935.
47. Tian, J.; Zhao, J.; Xu, Z.; Liu, B.; Pu, J.; Li, H.; Lei, Q.; Zhao, Y.; Zhou, W.; Li, X.; et al. Bioinformatics Analysis to Identify Key Invasion Related Genes and Construct a Prognostic Model for Glioblastoma. *Sci Rep* **2025**, *15*, 10773, doi:10.1038/s41598-025-95067-x.
48. Zhang, Y.; Zhou, Y.; Zhang, S.; Zhou, L. Expression Profile and Prognostic Relevance of Immune Infiltration-Related RBMS1 in Gliomas: A Multidimensional Integrative Analysis. *J Cancer Res Clin Oncol* **2025**, *151*, 205, doi:10.1007/s00432-025-06254-2.
49. Li, G.; Zhao, Y.; He, Y.; Qian, Z.; Liu, Y.; Li, X.; Li, L.; Liu, Z. Machine Learning-Based Construction of Immunogenic Cell Death-Related Score for Improving Prognosis and Personalized Treatment in Glioma. *Sci Rep* **2025**, *15*, 30417, doi:10.1038/s41598-025-15658-6.
